# LFA-1 antagonist (BIRT377) similarly reverses peripheral neuropathic pain in male and female mice with underlying sex divergent peripheral immune proinflammatory phenotypes

**DOI:** 10.20517/2347-8659.2019.18

**Published:** 2019-07-22

**Authors:** Shahani Noor, Melody S. Sun, Arden G. Vanderwall, Mara A. Havard, Jacob E. Sanchez, Nathan W. Harris, Monique V. Nysus, Jeffrey P. Norenberg, Harrison T. West, Carsten R. Wagner, Lauren L. Jantzie, Nikolaos Mellios, Erin D. Milligan

**Affiliations:** 1 Department of Neurosciences, School of Medicine, University of New Mexico, Albuquerque, NM 87131, USA.; 2 Department of Anesthesiology and Critical Care, University of New Mexico, Albuquerque, NM 87131, USA.; 3 Department of Pediatrics and Neurology, Johns Hopkins University School of Medicine, Baltimore, MD 21205-2196, USA.; 4 Department of Radiopharmaceutical Sciences, College of Pharmacy, New Mexico Center for Isotopes in Medicine, University of New Mexico, Albuquerque, NM 87131, USA.; 5 Department of Medicinal Chemistry, University of Minnesota, College of Pharmacy, Minneapolis, MN 55455, USA.

**Keywords:** Neuropathic pain, glia, neuroimmune, peripheral immune, T cells

## Abstract

**Aim:**

The majority of preclinical studies investigating aberrant glial-neuroimmune actions underlying neuropathic pain have focused on male rodent models. Recently, studies have shown peripheral immune cells play a more prominent role than glial cells in mediating pathological pain in females. Here, we compared the onset and duration of allodynia in males and females, and the anti-allodynic action of a potentially novel therapeutic drug (BIRT377) that not only antagonizes the action of lymphocyte function-associated antigen-1 (LFA-1) to reduce cell migration in the periphery, but may also directly alter the cellular inflammatory bias.

**Methods:**

Male and female mice were subjected to peripheral nerve injury chronic constriction injury (CCI) applying two methods, using either 4–0 or 5–0 chromic gut suture material, to examine potential sex differences in the onset, magnitude and duration of allodynia. Hindpaw sensitivity before and after CCI and application of intravenous BIRT377 was assessed. Peripheral and spinal tissues were analyzed for protein (multiplex electrochemiluminescence technology) and mRNA expression (quantitative real-time PCR). The phenotype of peripheral T cells was determined using flow cytometry.

**Results:**

Sex differences in proinflammatory CCL2 and IL-1β and the anti-inflammatory IL-10 were observed from a set of cytokines analyzed. A profound proinflammatory T cell (Th17) response in the periphery and spinal cord was also observed in neuropathic females. BIRT377 reversed pain, reduced IL-1β and TNF, and increased IL-10 and transforming growth factor (TGF)-β1, also an anti-inflammatory cytokine, in both sexes. However, female-derived T cell cytokines are transcriptionally regulated by BIRT377, as demonstrated by reducing proinflammatory IL-17A production with concurrent increases in IL-10, TGF-β1 and the anti-inflammatory regulatory T cell-related factor, FOXP3.

**Conclusion:**

This study supports that divergent peripheral immune and neuroimmune responses during neuropathy exists between males and females. Moreover, the modulatory actions of BIRT377 on T cells during neuropathy are predominantly specific to females. These data highlight the necessity of including both sexes for studying drug efficacy and mechanisms of action in preclinical studies and clinical trials.

## INTRODUCTION

While male and female rodent models of peripheral neuropathic pain generate similar clinical features such as pathological sensitivity to light touch referred to as allodynia, emerging evidence suggests that the biochemical and cellular aspects underlying allodynia are different between the sexes. Clinical evidence strongly implicates sex differences in pain sensitivity^[1,2]^, and preclinical data supports these clinical findings by demonstrating that peripheral immune and glial cells exhibit sex differences in response to peripheral nerve injury leading to neuropathy^[3–6]^. Understanding sex divergent components of pain pathophysiology has drawn significant attention and is of paramount importance for identifying effective pain therapeutics in males and females.

Chronic neuropathic pain following peripheral nerve injury involves dynamic neuroimmune interactions between peripheral immune cells that traffic to the injured nerve, the dorsal root ganglia (DRG), and the spinal cord, and the actions of glial cells within the spinal cord^[7–9]^. Peripheral nerve injury leads to alterations in proinflammatory cytokines including interleukin (IL)-1β, IL-6, and TNF, and anti-inflammatory cytokines such as IL-10 and transforming growth factor-β (TGF-β1) at anatomical sites of the pain pathway^[10–16]^. Spinal glia and DRG satellite glia contribute to persistent allodynia by responding to and releasing these proinflammatory cytokines and by reducing IL-10 expression^[12,14,17–19]^. Additionally, the chemokine CCL2 is elevated in DRG of injured nerves and facilitates leukocyte migration to DRG and spinal cord^[19,20]^. However, most of these reports were studied male models of neuropathy or the sex was unspecified, with few studies utilizing females^[21–24]^, or compared sex differences underlying pathological pain^[3–5,25–28]^.

A substantial role of the adaptive immune response is now recognized as underlying aberrant neuroimmune actions following nerve injury. Activated CD4 T cells, specifically proinflammatory Th1 and Th17 cells, infiltrate the injured peripheral nerve and the lumbar spinal cord (LSC) ^[29–33]^, and are thought to contribute to glial activation^[34]^. Conversely, anti-inflammatory T regulatory (Treg) cells control spinal glial-immune proinflammatory activation and are protective against neuropathy^[35]^. A few studies have implicated a T cell role by examining cell migration to the DRGs^[27,36]^ or the spinal cord^[30]^ in neuropathic females. However, it is critical to identify T cell subtypes present in these key anatomical regions because discrete subtypes exert a distinctly different impact on surrounding tissue during chronic pain. The critical roles of subtypes of T cells within discrete anatomical pain-related regions (peripheral or central) remain unclear.

We hypothesized that peripheral immune and glial responses following peripheral nerve damage are quantitatively and qualitatively (specific immune cells) different between sexes. If true, pain therapy that targets specific immune actions may require distinctly different mechanisms to exert efficacy. Lymphocyte function-associated antigen-1 (LFA-1) is an adhesion molecule expressed on myeloid and T cells and possibly spinal microglia and is critical for immune cell adhesion and migration^[37]^. In addition to the widely characterized role of LFA-1, emerging evidence suggests that LFA-1 regulates various macrophage proinflammatory functions as well as T cell activation and differentiation^[38–43]^. Based on existing gaps in understanding sex differences in glial, innate or adaptive immune cell function driving neuropathic pain and the related cytokine/chemokine repertoire, the current study examined whether: (1) the development, magnitude, and duration of mechanical allodynia were different between male and female mice subjected to a well-established peripheral nerve injury (CCI) model; (2) blocking immune cell migration and/or altering the proinflammatory phenotype by preventing peripheral LFA-1 actions using a blood-spinal barrier impermeable small molecule antagonist, BIRT377 ^[42,44,45]^, reduces allodynia in males and females; and (3) BIRT377 directly modulates myeloid/glial-derived and T cell-related pro- and anti-inflammatory cytokine expression. The results identified differences in the magnitude of immune factor expression contributing to neuropathy between males and females, and that BIRT_337_ reversed allodynia similarly between males and females, and altered the corresponding expression of sex-specific immune factors.

## METHODS

### Animals

Experiments were performed using 10–14 week-old C57BL/6 mice (wildtype; FFID: IMSR JAX:000664) purchased from Jackson Laboratories or were bred in-house with parent mice purchased from Jackson Laboratories (Bar Harbor, ME, USA). Age at the time of surgery ranged from 11–12 weeks for males and 10–12 weeks for females. Mice were housed with their cage-mates in groups of 2–5, in temperature (23 °C ± 2 °C) and light (12:12 light:dark; lights on at 6:00 am) controlled rooms, fed standard rodent chow and water *ad libitum,* and acclimated for 1–2 weeks prior to handling. All mice were routinely monitored by the animal care staff under the direction of the institutional veterinarian, with cages and bedding changed every 7 days. Mice were maintained in separate male or female mouse colonies and were behaviorally assessed in separate testing rooms at the University of New Mexico (UNM) Health Sciences Center (HSC) Animal Facility. Pilot studies of behavioral hindpaw threshold responses were conducted to determine whether different phases of the estrous cycle altered behavioral outcomes at baseline (BL) and after surgical manipulation. Despite female mice entering experiments at different phases of the estrous cycle, hindpaw responses remained stable and predictable. Consequently, the stage of the estrous cycle varied and was not considered a key factor influencing hindpaw sensory responses throughout the chronic neuropathy paradigm.

All procedures were approved by the Institutional Animal Care and Use Committee of the UNM HSC, conducted in accordance to the NIH Guidelines for the Care and Use of Laboratory Animals, and closely adhered to recommendations from the International Association for the Study of Pain for the use of animals in research (Foundation for Biomedical Research, The Biomedical Investigator’s Handbook for Researchers Using Animal Models. Washington, D.C.: FBR, 1987. WWW: http://www.fbresearch.org/).

### Chronic constriction injury

A modification of Bennett and Xie’s^[46]^ sciatic nerve (SCN) Chronic constriction injury (CCI) was used after BL hindpaw threshold assessment, as detailed previously^[47]^. Briefly, following isoflurane anesthesia (induction at 3.0 vol.% followed by 1.5 vol.%−2.0 vol.% in oxygen, 2.0 L/min), the dorsal left thigh was shaved and cleaned using 70% Ethanol (EtOH) that was air dried prior to surgery. Using aseptic procedures, the SCN was exposed using blunt dissection scissors through the muscular fascia. Sterile plastic probes were used to locate and lift the SCN from its position between the muscles. Three, 2 cm-length pieces of chromic gut suture (Ethicon: 4–0 or 5–0, Cat#635H and Cat#634G, respectively) were then snugly tied around the SCN proximal to the trifurcation with care to avoid pinching the nerve, with ~1.5 mm spacing between sutures. Throughout this process, the nerve was kept thoroughly irrigated using isotonic sterile saline (Hospira; Cat#NDC 0409–4888-03). Sham surgeries were performed identically, but without the chromic gut ligation. The nerve was then placed back into its position using the plastic probes, and the muscles were then closed using one 4–0 silk suture (Ethicon; Cat#83G). Skin was closed using two Reflex^™^ wound clips (Kent Scientific Corp; Cat#INS750344). Total time for the surgical procedure was ~20 min, followed by a ~10 min recovery from anesthesia. Body weight was monitored prior to and after surgery to confirm healthy recovery [Supplementary Figure]. Following surgery, wound condition, hindpaw autotomia, activity levels and grooming appearance were checked routinely (each 1–2 days). Less than 1.0% of animals revealed abnormal recovery and were immediately euthanized when identified.

### Behavioral assessment of hindpaw mechanical allodynia

Mechanical allodynia was chosen for investigation because pathological pain intensity (touch sensitivity) occurs clinically at much lower ranges of stimulus intensity compared to that observed when examining mechanical or thermal hyperalgesia. Thus, the impact of clinical allodynia is thought to be much greater^[48]^. Mice were habituated to the testing environment for ~45 min within the first 4 h of the light cycle, for four periods over the course of one week prior to BL hindpaw assessment. Hindpaw threshold responses to light mechanical stimuli were assessed by adopting principles of the von Frey fiber test originally developed for the rat^[49]^, and modified for the mouse, as recently described in detail^[47]^. Hindpaw assessment occurred within the first 2 h of the light cycle in testing groups of 4–6, with testers blind to experimental conditions. Each group comprised a minimum of 1–4 mouse/mice per condition to ultimately reach *n* = 6 mice/experimental condition. Time points for behavior assessments were chosen based on pilot studies and prior reports to capture potential subtle differences during the development of allodynia and BIRT377-mediated pain reversal.

The von Frey test was applied using nine calibrated monofilaments (touch-test sensory evaluator: North Coast Medical; Cat#NC12775) applied for a maximum of 3.0s to the plantar surface of both the left and right hindpaws, with laterality of hindpaw testing occurring randomly, with repeated stimulus presentations to a single animal using a minimum inter-trial stimulus period of 30s. The log intensity of the nine monofilaments used is defined as log_10_ (grams × 10,000) with the range of intensity being as follows, reported in log (grams): 2.36 (0.022 g), 2.44 (0.028 g), 2.83 (0.068 g), 3.22 (0.166 g), 3.61 (0.407 g), 3.84 (0.692 g), 4.08 (1.202 g), 4.17 (1.479 g), and 4.31 (2.042 g). Testing began using the fiber marked 3.22 with subsequent monofilaments used based on the response/non-response of the mouse to the previous monofilament tested: if no response was elicited by the monofilament stimulus presented (e.g., 3.22), the next “heavier” monofilament was tested (e.g., 3.61); if a response was elicited by the monofilament stimulus presented (e.g., 3.22), the next “lighter” monofilament was tested (e.g., 2.83). A maximum total of six stimulus presentations were applied to each paw. The total number of positive and negative responses were then entered into the computer software program, PsychoFit (http://psych.colorado.edu/~lharvey:RRID:SCR_015381) to determine the absolute withdrawal threshold (50% paw withdrawal threshold), as previously described^[50]^. The PsychoFit program fits a Gaussian integral psychometric function to the observed withdrawal rates for each monofilament using a maximum-likelihood fitting method^[47,51]^. The interpolated 50% withdrawal thresholds were then used for statistical analysis.

### BIRT377 preparation

(*R*)-5-(4-bromobenzyl)-3-(3,5-dichlorophenyl)-1,5-dimethylimidazolidine-2,4-dione (BIRT377) was first reported and characterized by Kelly *et al.*^[45]^. BIRT377 is a small molecule that blocks the active conformational change of the transmembrane β_2_-integrin adhesion molecule, leukocyte function-associated antigen-1 (LFA-1), that is expressed on leukocytes (e.g., T cell and myeloid cells)^[37]^. Upon activation from chemotactic signaling, LFA-1 undergoes a series of conformational changes from a bent inactive position to a progressively straightened and active position, thus allowing binding of LFA-1 with the surface receptor, intercellular adhesion molecule-1 (ICAM-1) expressed on endothelial cells^[52]^. Upon LFA-1/ICAM-1 interaction, cells expressing LFA-1 (leukocytes) are capable of undergoing transendothelial migration, and subsequently traffic to regions where damage- or pathogen-associated tissue signals arise. Therefore, BIRT377 binding to LFA-1 inhibits LFA-1/ICAM-1 molecular interactions, and prevents circulating leukocyte cell adhesion and migration^[44,45,53]^ to sites of inflammation. BIRT377 abolishes T cell and antigen presenting cell interactions (referred as immune synapse), which is crucial for T cell activation^[54]^. Moreover, BIRT377 is bioavailable and easier to formulate for oral administration than antibodies against LFA-1^[44,45]^ and BIRT377 is impermeable to blood-spinal cord barrier^[42]^. Therefore, i.v. injection of BIFT377 is expected to impact: (1) leukocyte migration to peripheral sites and across spinal endothelial cells; (2) macrophage proinflammatory function and possibly T cell differentiation in the periphery; and (3) neuron-to-glial and immune communication in the LSC due to BIRT377-mediated modulation in the periphery during neuropathy.

In an initial experiment, BIRT^377^ was gifted by HTW and CRW (University of Minnesota, College of Pharmacy, MN, USA), with later experiments where BIRT377 was made commercially available (Tocris; Cat#4776). BIRT377 was initially reconstituted in 200 proof ethyl alcohol EtOH (Sigma-Aldrich; Cat#7023) as a stock solution (22.156 mg/mL), followed by creating aliquots (221.56 μg of BIRT377 in 10 μL), which were stored in a clean sealed container at 4 °C for later use. On the day of intravenous (i.v.) injection, one aliquot was diluted using sterile water (Hospira; Cat# NDC 0409–4887-10), such that each 50 μL i.v. injection contained 2.5 μg BIRT377 (113.089 μM), and vortexed for 2 min. This dose was chosen based on a pilot study of various doses (ranging from i00 ng to 5 μg) that 2.5 μg was the lowest reliably efficacious dose in rats (unpublished data and^[42]^). Vehicle contained 0.226% EtOH in sterile water. Animals were injected within the hour following BIRT377 dilution.

### Intravenous BIRT377 injection

For all experiments characterizing BIRT377 efficacy, i.v. BIRT377 or equivolume vehicle injection into tail veins of unanesthetized mice occurred on Day 10 post-surgery within 2.5 h of the initiation of the light cycle. Using aseptic procedures, 50 μL of BIRT377 or vehicle was collected into individual 1 cc, 27-G 5/8 insulin syringes (Becton Dickinson; Cat#329412). The weight of each mouse was recorded followed by placement for 30 s under a heat lamp with the mouse held in place by the tail and a soft, clean cloth placed over the body to avoid excessive heat to the body while leaving the tail exposed. Heating the tail facilitates tail vein dilation for ease of injection. Each mouse was moved immediately into a plastic restraint with a slit through the top and back so as to allow easy placement of the mouse into the restraint, and proper positioning of the tail. Held firmly in place, a 27-G sterile needle attached to a sterile 1 cc syringe was inserted into the lateral tail vein, followed by a small amount of blood efflux into the syringe hub, with a subsequent 5 s injection. Success of achieving accurate needle placement upon the first attempt was greater than 99%. Following injection, a small piece of sterile gauze was placed over the injection site to stem bleeding, and the mouse was removed from the restraint and placed back into its home cage. All mice appeared normal (e.g., moving, grooming, interacting) following injections. The total time required for handling and injection was less than 2 min without anesthesia.

### Sciatic nerve biopsy

Following characterization of the timecourse of hindpaw sensory thresholds from sham or CCI treated mice using either 4–0 or 5–0 chromic gut, the presence of suture material and the condition of the SCN were carefully examined. Following complete return of sensory thresholds similar to BL levels, the ipsilateral SCN was dissected, and the degree of both suture absorption and nerve perturbation was noted. The appearance of each SCN at the time of dissection was documented by photograph.

To accomplish biopsies, animals were euthanized just prior to biopsy using 2% CO_2_ in a closed container followed by cervical dislocation. An incision was then made on the ipsilateral skin overlaying the CCI manipulation, and the skin was retracted to expose the underlying muscle and surrounding area, which appeared to be healthy. Blunt dissection scissors were used to re-expose the underlying SCN. The surrounding muscle and remaining sutures and encapsulating sheath were removed, followed by dissection of an approximately 1 cm length of SCN. The nerve segment was then placed next to a ruler (in centimeters), on a clean black surface for documentation.

### Tissue collection for RNA and protein analysis

Tissue collection was conducted in six cohorts of mice (8 mice in each cohort, N of 1 from each experimental condition) as previously described^[47,51]^ and modified as described here. Immediately following behavioral analysis on Day 13 post-surgery (Day 3 post-injection), mice were deeply anesthetized under isoflurane (10 min in 5% isoflurane and in oxygen at 2 L/min), followed by rapid transcardial perfusion with ice cold 0.1 M phosphate buffered saline (PBS; pH = 7.4; flow rate 10 mL/min). Following collection of the spleen, mice were placed on a frozen gel refrigerant pack (Glacier Ice, Pelton Shepherd Industries), and the LSC (L3-L6) was dissected, with the dorsal spinal cord ipsilateral and contralateral to the sciatic ligation stored separately. Additionally, lumbar DRG (L4-L6) ipsilateral to the sciatic ligation, and the SCN were dissected. All tissues were immediately placed in DNase/RNase/Protease-free 1.5 mL disposable pellet mixer microtubes (VWR International; Cat#47747–358), briefly spun down, frozen on dry ice, and stored at −80 °C for future analysis.

### Total RNA isolation

Total RNA was extracted as described previously^[47]^, with minor modifications as briefly described here. Extraction was performed using the miRNeasy Micro Kit (Qiagen; Cat#217084) per manufacturer’s instructions except where noted. Homogenization was performed using a motormlized VWR disposable pellet mixer and cordless motor pestle system (VWR; cordless pestle motor: Cat#47747–370; 1.5 mL microtubes: Cat#47747–362; 1.5 mL pestle: Cat#47747–358; and 1.5 pestle and microtube combo: Cat#47747–366: DRGs only) followed by addition of Qiazol Lysis Reagent (Qiazol; Qiagen; Cat#79306). DRGs were then transferred into microtubes prior to homogenization. Samples were homogenized in Qiazol with the motorized pestle for 60 s, and used for RNA extraction (Qiagen; miRNeasy Micro Kit). SCN and LSC were homogenized prior to aliquoting the tissue into two microtubes for protein or RNA extraction. 100 μL of chilled 1 × phosphate buffered saline (PBS; 10 × PBS diluted to 1 × with DNase/RNase free water; Sigma-Aldrich; Cat#P7059; Cat#W4502 respectively) was added to the tube containing the tissue. The SCN was chopped quickly using scissors for 30 s. Both SCN and LSC were then homogenized with the motorized pestle for 15 s. After initial homogenization, 40 μL of the homogenized solution was removed and placed into a separate 1.5 microtube containing 150 μL of chilled Qiazol and homogenized for an additional 15 s for LSC, or 30 s for SCN, prior to using the miRNeasy Micro Kit for RNA extraction.

Minor changes were incorporated for mRNA extraction using the miRNeasy kits as follows. An initial homogenization in 150–200 μL of Qiazol occurred, with the final volume increased to 700 μL following homogenization. Samples were vortexed and incubated at room temperature (RT) for 7 min, followed by the addition of 140 μL of chloroform (Sigma-Aldrich; Cat#C_2432_). The samples were then hand-shaken vigorously for 15 s, incubated for 4 min at RT, hand-shaken vigorously for 10 s, and then centrifuged in 4 °C at 12,000 × *g* for 15 min. A portion, 300 μL, of the aqueous layer was extracted and placed into a clean RNase/DNase/Protease free 1.5 mL tube and 1.5 × aqueous layer (450 μL) of 200 proof EtOH (Sigma-Aldrich; Cat#E7023) was added to tube, pipetted 4–6 × to mix, moved to collection columns, and centrifuged in ~20 °C at 9,000 × *g* for 30 s. This was followed by a wash of 700 μL of RWT (provided with Qiagen kit) and centrifuged (~20 °C at 9,000 × g, 30 s), washed 2 × with 500 μL RPE (provided with Qiagen kit) and centrifuged (~20 °C at 9,000 × *g*, 30 s) after each, and washed 2 × with 500 μL 80% EtOH (100% EtOH diluted with sterile RNase/DNase/Protease free water; Sigma-Aldrich; Cat#W4502), and centrifuged (~20 °C at 9,000 × *g*, 2 min) after each. Caps were cut from columns and samples were dried by centrifugation (~20 °C at 20,627 × *g*, 12 min), and placed into RNA collection tubes with 14 μL sterile water (provided with Qiagen kit) added directly to the column filter, and centrifuged (~20 °C at 20,627 × *g*, 1 min). The concentration and quality of the total RNA was assessed by NanoDrop (Thermo Scientific, MA, USA).

### mRNA Analysis by Quantitative Real-Time PCR

Total RNA samples were diluted to a standardized RNA concentration: 90 ng/μL for SCN, 70 ng/μL for lumbar dorsal horn, and 100 ng/μL for DRG. Total RNA (0.9–1.2 μg) was used to synthesize cDNA. For reverse transcription (cDNA), SuperScript^™^ IV VILO^™^ cDNA Synthesis Kit (Invitrogen) was used per manufacturer’s instructions. Levels of mRNA transcripts were measured and analyzed, as previously described^[47,55]^. The following dilution factors (indicated in parentheses) were applied to cDNA samples for assessment of transcripts of interest in given tissues: ipsilateral and contralateral LSC (1:2.2), ipsilateral SCN (1:2.5), and ipsilateral DRG (1:3). The 1:200 dilutions of cDNA were used for assessment of the normalizer transcripts (18s RNA) for each of the tissue samples. Levels of mRNAs as well as 18s rRNA (*Rn18s*) were assayed in triplicate via quantitative real-time PCR (qRT-PCR) with Taqman Gene Expression Assays (cat# 4351370, ThermoFisher Scientific). In cases of triplicates with standard deviation of more than 0.1, the average value of the two closest replicates were included. All selected gene expression assays were identified by the manufacturer to be the “best coverage” assays, unless otherwise noted, and designed to exclude detection of genomic DNA. mRNA levels were analyzed with the formula: $\text{C}=2^{CT}{}^{{}^{Normalizer}}/2^{CT^{Target}}$, as previously described^[55,56]^.

To test whether BIRT377 treatment influenced the inflammatory milieu in collected tissues, the following pain-relevant proinflammatory and anti-inflammatory factors were assessed: C-C motif chemokine ligand 2 (CCL2, *Ccl2*), interleukin-1β (IL-1β, *il1b*), (TNF, TNFα, *Tnf*), interleukin-10 (IL-10, *Il-10*), TGF-β1, *Tgfb1.* Monocyte and T cell-specific cytokines and cellular markers were analyzed: integrin alpha M (CD11b, *Itgam*, a common monocyte/macrophage marker), cluster of differentiation 3 (CD3; expressed on all T cells), forkhead box P3 (FOXP3, *Foxp3*) which is expressed by Treg cells, and Interleukin-17a (IL-17A, *Il-17a*, expressed by proinflammatory Th17 cells)^[57–60]^. To assess whether BIRT377 treatment may lead to allodynia reversal by modulating glial activation in the LSC, the transcript levels of the microglial specific marker, transmembrane protein 119 (TMEM119, *Tmem119*)^[47,61]^ and the astrocyte activation marker, glial fibrillary acidic protein (GFAP) were evaluated. All 48 samples (96 for ipsilateral and contralateral LSC) and a “no template control” sample for each tissue type was processed for the cDNA preparation or real-time PCRs simultaneously.

### Multiplex determination of splenic cytokine and chemokine expression

Frozen spleens were homogenized for 60 s in a buffer with protease inhibitors (MesoScale Discovery) while kept on ice, and subsequently sonicated (settings: 5 pulses, at 50%, Fisher Scientific). Tissue samples were then centrifuged at 4200 × *g* at 4 °C for 10 min to pellet cellular debris. Cellular lysates (supernatant) were collected in a new set of tubes, and protein concentrations were measured by Quickstart^™^ Bradford Protein Assay Kit (Biorad, CA, USA). Splenic cytokine and chemokine levels were determined using V-Plex^™^ multiplex immunoassays (MesoScale Discovery), as described previously^[47,51,62,63]^. Briefly, calibrators (provided by the kit) or samples (100 μg protein from each experimental sample per well) were loaded onto a “multi-spot” plate in duplicates. Each plate-well is pre-coated with antigen-specific “capture” antibodies on independent, spatially well-defined “spots” that are in turn connected to a working electrode surface. Following incubation with protein lysates, immobilized proteins were recognized by SULFO-TAG^™^-conjugated antigen-specific “detection” antibodies. Samples were read using a Quickplex SQ120 Imager (MesoScale Discovery).

### Preparation of Naïve CD4 T cell suspension

To further investigate whether LFA-1 contributes to T cell differentiation and their functional responses, CD4 T cells were cultured with or without BIRT377 (500 ng/mL). A total of 20 mice (wildtype, FFID: IMSR_JAX:000664; 10 females and 10 males, 8–10 week-old) were compared in this study. In each experiment, 5 male and 5 female mice were used, with two repeat experiments (total of 10 male and 10 female mice). No handling occurred with these mice. Mice were sacrificed with CO_2_ asphyxiation, followed by cervical dislocation. Under sterile conditions, spleens and lymph nodes (cervical, inguinal and brachial) were collected in tubes containing ice-cold PBS with 2% fetal bovine serum (FBS; Gibco, Thermofisher Scientific, MA, USA). Spleens and lymph nodes were disrupted using a micro-plunger to press the tissues through a 70 μm nylon mesh. Cells were centrifuged at 300 × *g* for 10 min at 4 °C and resuspended at 1 × 10^8^ nucleated cells/mL in PBS with 2% FBS and 1mM EDTA (ethylene diaminetetraacetic acid). Naïve CD4 T cells (CD4^+^CD44^low^CD62L^high^) were isolated using EasySep^™^ Naïve CD4 T Cell Isolation Kit, per manufacturer’s instructions (Stemcell Technologies, BC, Canada). In this technique, non-naïve T cells were labeled with biotinylated antibodies and magnetic particles, allowing for the collection of desired naïve T cells using an EasySep^™^ magnet (Stemcell Technologies, BC, Canada). Live cells were counted on a hemocytometer.

### CD4 T cell culture with BIRT377

Our prior data demonstrated that BIRT377 (500 ng/mL) induces a switch of stimulated macrophage (RAW264.7) from a proinflammatory bias to an anti-inflammatory state^[42]^. To examine effects of blocking LFA-1 actions on T cells *in vitro,* isolated male or female derived CD4 T cells were resuspended with complete RPMI media^[51]^ and treated with BIRT377 (500 ng/mL). A 24-well tissue culture plate was pre-coated with anti-CD3 antibody (10 μg/mL in sterile PBS; R&D systems), and stored overnight at 4 °C. The tissue culture plate was pre-warmed and washed 3 × with sterile PBS before use. One million CD4 T cells were plated per well, with 2–3 well-replicates per experimental condition. Cells were cultured with either Th17 or Treg differentiation conditions, as described previously^[64,65]^, with minor modifications. Briefly, in the presence of TCR (T cell receptor) stimulation by anti-CD3 antibody, a cocktail of Th17 polarizing cytokines was applied as follows: anti-mouse CD28 (5 μg/mL), TGF-β1 (2.25 ng/mL), IL-1β (20 ng/mL), IL-6 (30 ng/mL), IL-23 (30 ng/mL), anti-IFNγ (10 μg/mL). For Treg differentiation, a cocktail containing CD28 (2 μg/mL), IL-2 (20 ng/mL), and TGF-β1 (5 ng/mL) was added to the cells. BIRT377 treatment (500 ng/mL) was applied simultaneously with the Th17 or Treg cytokine T cell stimulation mixtures throughout the culture timecourse (4 days). Anti-CD3, anti-CD28, and anti-IFNγ were purchased from eBioScience (Thermofisher Scientific, MA, USA), and TGF-β1, IL-1β, IL-6, IL-23 and IL-2 were purchased form PeproTech (NJ, USA). Pooled CD4 T cells from naïve mice (5 male or 5 female mice maintaining sex-separate tubes) were used for two independent experiments, followed by flow cytometry procedures similar to that detailed in prior reports^[66–69]^.

### Intracellular staining and flow cytometry

On Day 4 of T cell differentiation culture, cells were collected, washed with 1 × PBS (300 × *g,* 5 min, 4 °C), kept on ice to prevent cytokine secretion, and stained for intracellular levels of pro- or anti-inflammatory cytokine or transcription factor (protein) production. Proinflammatory markers included retinoid-related orphan receptor-γt (RORγt), the Th17-associated transcription factor, and the cytokines, IL-17A and TNF. Anti-inflammatory markers included the cytokines IL-10 and TGF-β1. Cells were first stained for viability, surface antigens, and intracellular proteins, as described previously, with minor modifications^[51]^. Briefly, cells were pelleted into FACS staining tubes (BD Falcon) and incubated with Fc-block (blocking buffer) for 10 min on ice, stained with viability dye (25 min, on ice, dark) and then incubated with CD4 antibody (25 min, on ice, dark). To stain for RORγt and intracellular cytokines (IL-17, TNF, IL-10 and TGF-β1), an intracellular cytokine staining kit (Cat#00–5523-00, eBioScience, Thermofisher Scientific, MA, USA) was used. With this protocol, cells were fixed and permeabilized for 60 min at RT and protected from light. Then cells were washed 2× with permeabilization buffer (2 mL/tube) for 5 min at RT. To prevent non-specific binding of the antibodies, cells were incubated with blocking buffer (containing 2.5 μg anti-mouse CD32 purified antibody) for 15 min on ice. Without washing, fluorochrome conjugated antibodies against RORγt, IL-17A, and TNF (cells from Th17 differentiation wells), or against IL-10 and TGF-β1 (cells from Treg differentiation wells), were added, and cells were incubated for 45 min at RT in the dark. Cells were then washed 2 × again with 2mL of permeabilization buffer at 300 × *g* for 5 min, at 4 °C, resuspended in 300 μL FACs buffer (1 × PBS containing 1% bovine serum albumin and 1mM EDTA), and kept on ice protected from light until data acquisition. Blocking buffer was purchased from BD Biosciences (Fc block, Cat#553141). All the fluorochrome conjugated antibodies were purchased from eBioscience (Thermofisher Scientific, MA, USA) and used at 0.125–1 μg/per 10^6^ cells per tube, as recommended by the manufacturer. T cell events (50,000) were collected using a BD LSR Fortessa Cell Analyzer, and later analyzed via FlowJo software v.8.7.4. Only viable (based on light scatter properties and viability dye) CD4 T cells (based on positive staining of surface CD4 antigen) were included for further analysis. Positive staining for transcription factor and/or cytokines were determined based on staining with fluorochrome conjugated isotype controls (IgG2b and IgGa).

### Experimental design and statistical analysis

**Figure 1.**
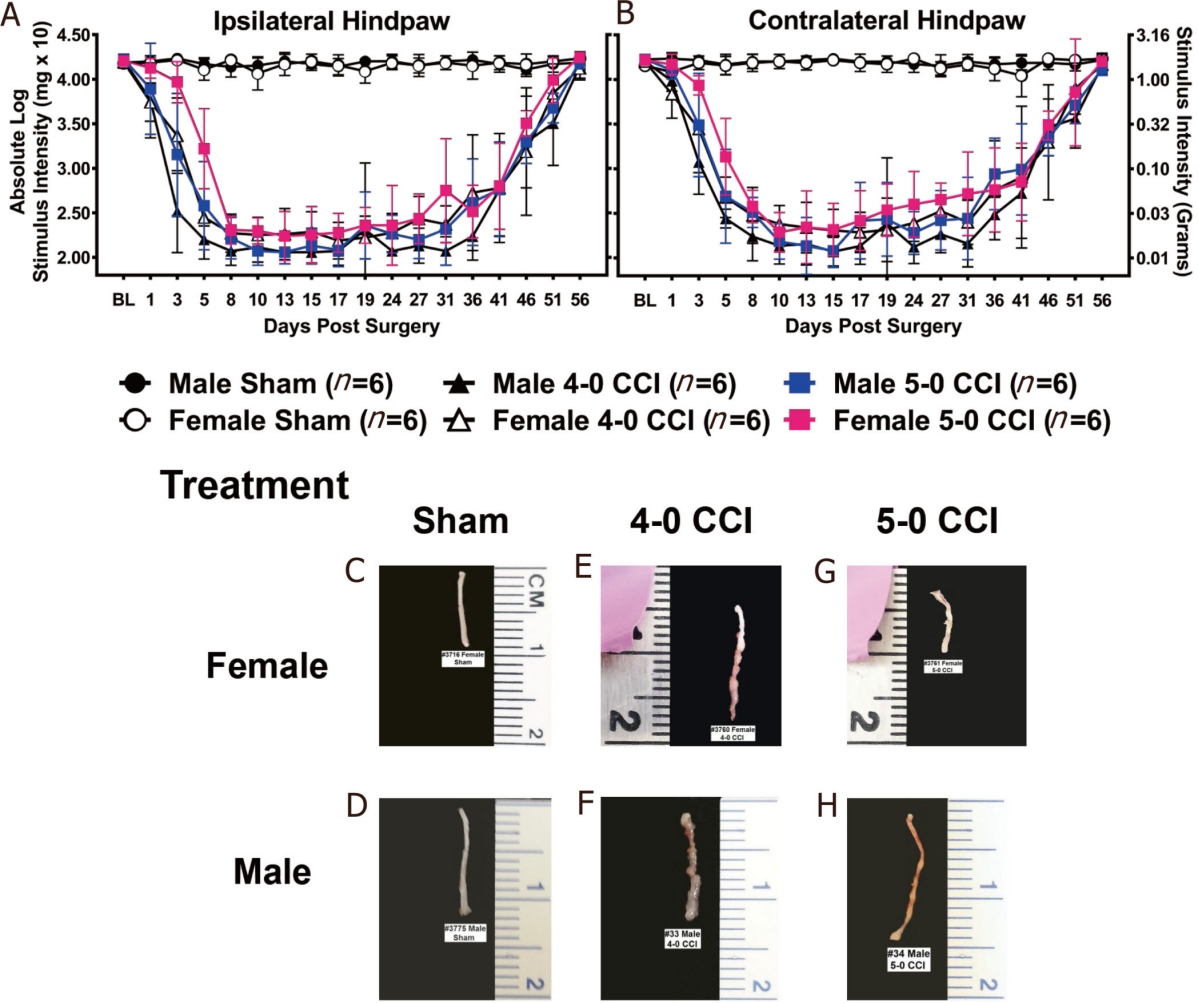
The timecourse of CCI-induced allodynia comparing 4–0 or 5–0 suture material is similar in both males and females. No significant differences were observed between groups at baseline (BL) assessment for hindpaw threshold responses either (A) ipsilateral or (B) contralateral to the sciatic nerve injury. Compared to mice that underwent sham manipulations, all mice with either 4–0 or 5–0 CCI reached maximal bilateral allodynia by Day 8–10 post-surgery and remained stably allodynic through Day 36 post-surgery. Main effects of time on hindpaw responses was observed from BL to Day 8 in the ipsilateral side (F_2.4,74.7_ = 207.56, *P* < 0.001) or Day 10 in the contralateral side (F_2.7,81.5_ = 212.64, *P* < 0.001). A main effect of surgery (ipsilateral: Day 8 – 27: F_2,30_ = 591.25, *P* < 0.001; contralateral: Day 10 – 19: F_2,30_ = 352.59, *P* < 0.001) was observed from hindpaw responses showing stable allodynia. A gradual spontaneous reversal of hindpaw responses similar to BL values was evident by Day 56 post-surgery, as supported by a main effect of time (ipsilateral: Day 27 – 56: F_3.5,105.0_ = 113.37, *P* < 0.001; contralateral: Day 19 – 56: F_4.3,131.6_ = 91.55, *P* < 0.001). Interestingly, sex and suture size had an effect on hindpaw responses only during the onset of allodynia, as shown by the main effect of sex (ipsilateral: BL - Day 8: F_1,30_ = 13.05, *P* = 0.001; contralateral: BL - Day 10: F_1,30_ = 9.03, *P* = 0.005). (C-H) Sciatic nerves were biopsied on Day 56 post-surgery. Compared to Sham (C) female and (D) male sciatic nerves, (E) female and (F) male nerves with peri-sciatic 4–0 CCI revealed a translucent sheath and remaining suture material surrounding the injury site, combined with significant discoloration and indentation of the nerves. (G) Female and (H) male mice with peri-sciatic 5–0 CCI revealed diminished or lack of sheath, and minimal suture material surrounding the injury site, combined with far less discoloration and indentation of the nerves. *n* = 6 for all groups

Three independent behavioral experiments were conducted with an *n* = 6 mice in each group in each experiment. Prior work demonstrates *n* = 4–6 mice/experimental condition is sufficient to yield reliable group differences when examining similar endpoints^[3,4,13,33–35,47]^. The initial experiment was an examination of differences in hindpaw sensitivity between sexes following peri-sciatic manipulations (sham *vs.* 4–0 *vs.* 5–0 CCI) during a 56-days timecourse. Thus, a 2 × 3 repeated measures analysis of variance (ANOVA) was conducted, with hindpaw thresholds assessed at BL and re-assessed every 1, 2, 3, or 5 days after surgical manipulation until complete resolution of allodynia was observed. The behavioral profile of the mice presented in Figure 1 was predicted to inform parameters of Experiment 2. That is, to characterize the earliest maximal onset, stable maintenance and duration of allodynia in both male and female mice. For experiment 2, injection of BIRT377 was administered on Day 10 after surgery, and the efficacy and duration of reversal from allodynia by BIRT377 was assessed. Experiment 2 design was a 2 (male *vs.* female) × 2 (vehicle *vs.* BIRT377) and analyzed by a 2-way repeated measures ANOVA where hindpaw assessment occurred prior to and following BIRT377 treatment. The goal of Experiment 3 was to examine the biochemical profile (protein and mRNA) in discrete tissue systems at a time when BIRT377 exerts maximal efficacy on stable allodynia as determined by Experiment 2. Therefore, Experiment 3 design was a 2 (male *vs.* female) × 2 (sham *vs.* CCI) × 2 (vehicle *vs.* BIRT377) and analyzed by a 3-way repeated measures ANOVA, with re-assessment of hindpaw thresholds terminating at peak BIRT377 efficacy. At this time, spleen, SCN, DRG, ipsilateral and contralateral LSC tissues were dissected and processed.

All behavioral data was graphed in GraphPad Prism version 7.02 (GraphPad Software Inc.; RRID:SCR_002798). All statistics were run using IBM SPSS Statistics version 24 (IBM; RRID:SCR_002865). ANOVAs were performed for data collected at BL and on injection day. For all other behavioral timepoints, repeated measures ANOVA were performed to assess differences of group and timecourse between treatments. The assumption of sphericity was assessed using Mauchly’s Test of Spericity (α = 0.05) and, if the assumption of sphericity was violated (*P* > 0.05), the reported degrees of freedom and p-values were adjusted using the Greenhouse-Geisser correction to protect against Type I errors^[47,51]^. Fisher’s LSD test was applied for *post hoc* analysis. Relative mRNA transcript levels from qRT-PCR were analyzed using 3-way ANOVA on GraphPad PRISM version 7.02 or SPSS. To control the type I error rate during all multiple comparisons, Fisher’s LSD test (reported with adjusted P values) was applied for *post hoc* examination of possible group differences selected *a priori.* Within-group outliers were detected by Grubbs’ Test using the GraphPad QuickCalc Outlier Calculator (https://graphpad.com/quickcalcs/grubbsi/) with α = 0.05.

An *in vitro* tissue culture experiment (Experiment 4) was conducted using CD4 T cells isolated from naïve male and female mice to investigate the effects of BIRT377 on T cell activation and differentiation. No behavioral assessment was performed on these mice. Flow cytometry data from Experiment 4 were analyzed by 2-way ANOVA using Graphpad Prism and Fisher’s LSD test for *post hoc* comparisons. The threshold for statistical significance for all sets of multiple comparisons was set *a priori* to α = 0.05. All data are presented as the mean ± Standard Error of the Mean.

## RESULTS

### Male and female mice with CCI of the sciatic nerve using either 4–0 or 5–0 chromic gut suture material develop allodynia with similar onset, duration and spontaneous recovery

The mouse CCI model has been performed using a range of suture types and sizes^[4,47,70,71]^. Here, we examined the profile of allodynia using chromic gut suture material of two thickness characteristics (4–0 *vs.* 5–0), whereby the 4–0 suture material is thicker than the 5–0 suture material. Assessment for hindpaw light mechanical touch responses at BL revealed no difference between male or female mice (ipsilateral: F_5,30_ = 0.78, *P* = 0.576; contralateral: F_5,30_ = 2.37, *P* = 0.063) [Figure 1A and B].

Compared to mice that underwent sham manipulations, mice that underwent CCI surgery developed bilateral allodynia, which replicated similar experiments reported previously^[47,51,72–75]^. All mice with either 4–0 or 5–0 CCI reached maximal bilateral allodynia by Day 8 (ipsilateral) or Day 10 (contralateral) post-surgery, with main effects of time (ipsilateral: F_2.4,74.7_ = 207.56, *P* < 0.001; contralateral: F_2.7,81.5_ = 2i2.64, *P* < 0.001) and surgery (ipsilateral: F_2,30_ = 141.3, *P* < 0.001; contralateral: F_2,30_ = 420.18, *P* < 0.001), and an interaction between time and surgery (ipsilateral: F_4.9,74.7_ = 53.99, *P* < 0.001; contralateral: F_5.4,81.5_ = 56.28, *P* < 0.001). Compared to sham-treated mice, stable bilateral hindpaw sensitivity (allodynia) persisted in CCI-treated mice ipsilaterally (Day 8–27) and contralaterally (Day 10–19), as supported by a main effect of surgery (ipsilateral: F_2,30_ = 591.25, *P* < 0.001; contralateral: F_2,30_ = 352.59, *P* < 0.001). A gradual and spontaneous return to levels similar to BL was observed bilaterally with complete reversal occurring by Day 56, as supported by a main effect of time (ipsilateral: Day 27–56 post-surgery: F_3.5,105.0_ = 113.37, *P* < 0.001; contralateral: Day 19–56 post-surgery: F_4.3,1316_ = 91.55, *P* < 0.001) and surgery (ipsilateral: F_2,30_ = 151.37, *P* < 0.001; contralateral: F_2,30_ = 192.63, *P* < 0.001), and the interaction between time and surgery (ipsilateral: F_7.0,105.0_ = 27.86, *P* < 0.001; contralateral: F_8.7,131_._6_ = 21.52, *P* < 0.001) [Figure 1A and B]. While hindpaw responses between males and females were similar during most of the timecourse following surgery, differences during the initial phase of allodynia were observed. Statistical differences in the onset of allodynia were revealed between males and females, as supported by a main effect of sex (ipsilateral: BL-Day 10 post-surgery: F_1,30_ = 13.05, *P* = 0.001; contralateral: BL-Day 10 post-surgery: F_1,30_ = 9.03, *P* = 0.005), and between 4–0 and 5–0 chromic gut suture. That is, in comparison with other groups, males with 4–0 chromic gut suture material (thicker than 5–0) developed robust allodynia by Day 3 post-surgery, while females with 5–0 chromic gut suture material did not develop clear maximal allodynia until Day 8 post-surgery.

Reversal from allodynia prior to full reabsorption of the chromic gut suture material was observed in virtually all of the mice treated with CCI, regardless of the chromic gut suture thickness. Representative photographs of each treatment condition (with suture removed) are presented [Figure 1C-H]. Unpublished reports that examined reabsorption of 4–0 chromic gut from the SCN at Day 72 post-surgery in a rat model of CCI revealed variable degrees of reabsorption, and often observed a complete absence of chromic gut material, despite the presence of allodynia^[75]^. This further supports a published report that by Day 60 and 120 post-CCI in rats, the connective tissue capsule has been resorbed^[46]^. The current report sought to conduct gross morphological examination of the SCN in the mouse model of 4–0 and 5–0 chromic gut CCI following resolution of allodynia.

The data suggest that, while SCNs from sham-operated mice appear translucent with little discoloration [Figure 1C and D], the nerves treated with 4–0 chromic gut suture were found to possess a sciatic sheath/capsule surrounding the sutures, which was similar between male and female mice [Figure 1E and F]. However, SCNs from mice treated with a 5–0 chromic gut CCI revealed visibly less remaining suture material and less encapsulating sheath compared to SCNs treated with 4–0 CCI [Figure 1G and H]. Upon further dissection of the sheath and sutures away from the nerve in 4–0 and 5–0 CCI, marked indentations beneath the ligature in both conditions were observed. These observations suggest that reversal of allodynia from CCI in mice involves processes that are independent of the presence of the sutures. That is, the physiological response to peri-sciatic CCI is critical in the resolution of allodynia, and is not dependent on the presence of factors from the suture material itself.

### The LFA-1 antagonist BIRT377 reverses allodynia in male and female mice

**Figure 2.**
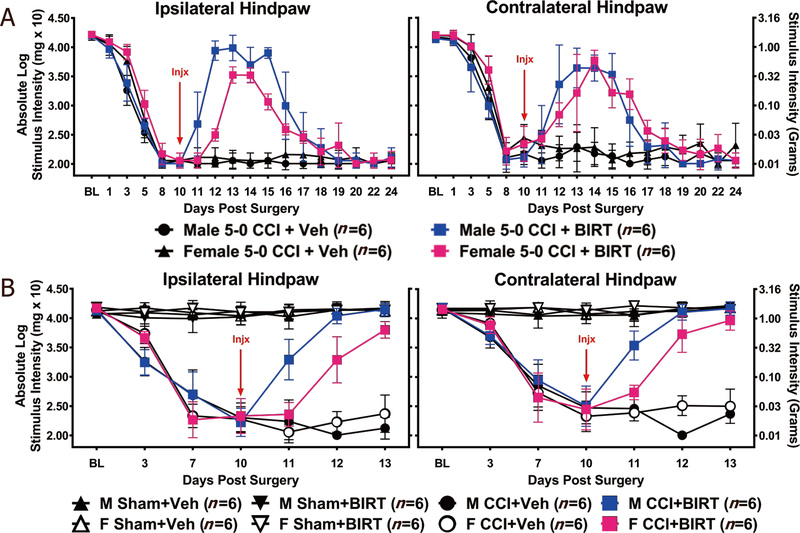
The LFA-1 antagonist, BIRT377, similarly reverses allodynia in males and females. Mice were either sham or treated with perisciatic 5–0 suture. (A) All groups of mice show similar BL threshold hindpaw sensitivity. Following 5–0 CCI, all animals develop clear allodynia during an 8-day timecourse, showing stable allodynia on Day 10, with a main effect of time (ipsilateral: F_3.02,60.48_ = 1003.02, *P* < 0.001; contralateral: F_3.56,71.15_ = 528.60, *P* < 0.001). Additionally, a main effect of sex during the development of allodynia is observed (ipsilateral: F_1,20_ = 21.27, *P* < 0.001; contralateral: F_1,20_ = 13.06, *P* = 0.002), with a significant interaction between time and sex (ipsilateral: F_3.02,60.48_ = 10.899, *P* < 0.001; contralateral: F_3.56,71.15_ = 3.23, *P* = 0.021). Following injections on Day 10 post-surgery, clear reversal from allodynia resulting from BIRT377 injection is observed compared to vehicle treated mice, supported by a main effect of injection (ipsilateral: F_1,20_ = 328.97, *P* < 0.001; contralateral: F_1,20_ = 74.47, *P* < 0.001). In addition, male mice treated with BIRT377 appeared to reverse from allodynia 1 day sooner than female BIRT377-treated mice, as observed in hindpaw responses ipsilateral (F_1,20_ = 12.12, *P* = 0.002) but not contralateral to the CCI, with an interaction between time and sex (ipsilateral: F_4.33,86.61_ = 9.33, *P* < 0.001; contralateral: F_4.92,98.34_ = 3.15, *P* = 0.012), and time and injection (ipsilateral: F_4.33,86.61_ = 70.29, *P* < 0.001; contralateral: F_4.92,98.34_ = 32.77, *P* < 0.001). (B) Experimental replication of BIRT377 reversal in males and females following CCI, with the onset and full development of bilateral allodynia occuring during a 10-day timecourse. Female mice reveal delayed onset of allodynia but no sex differences are observed by Day 10 post-surgery, when maximal allodynia is observed in both males and females. As previously observed, female 5–0 CCI mice treated with BIRT377 displayed slightly slower reversal from allodynia compared to males, with maximal bilateral reversal observed by Day 3 post-injection. *n* = 6 for all groups

Given the onset, intensity, and duration of allodynia was similar following either 4–0 or 5–0 peri-sciatic CCI in both males and females, subsequent experiments applied 5–0 chromic gut suture for CCI. Mice were assessed using the von Frey fiber test at BL, and no significant differences were observed. Mice with CCI developed maximal bilateral allodynia by Day 8 post-CCI [Figure 2A]. On Day 10 post-CCI, when all animals revealed stable and maximal allodynia, an i.v. injection of BIRT377 or vehicle was given followed by hindpaw re-assessment. Compared to mice given vehicle, complete reversal from allodynia was observed in both male and female animals following BIRT377 injection. Interestingly, a slight delay and duration of reversal of ipsilateral hindpaw sensitivity was observed in females compared to males. Specifically, BIRT377-mediated reversal of allodynia was delayed by 24 h in female mice, with allodynia returning 24 h earlier than their male counterparts. Contralateral hindpaw sensitivity was reduced by BIRT377 treatment to a similar degree and magnitude between males and females, as no statistical differences were observed. While it is clear that both male and female mice develop allodynia to the same degree with a similar duration, the difference in their response to i.v. BIRT377 suggests that the underlying processes leading to allodynia may not simply overlap, but instead may include distinct mechanisms between male and female mice.

In an effort to expand on characterizing potential sex-dependent differences in expression levels of peripheral immune signaling molecules (pro- and anti-inflammatory cytokines) during established peripheral neuropathy or BIRT377-induced reversal from neuropathy, a separate experiment was conducted to replicate the effect of BIRT377 on allodynia which was terminated at the peak of BIRT377 mediated pain reversal [Figure 2B] and tissues were collected for protein or mRNA (represented in subsequent figures) analysis. In this replication study, while differences in ipsilateral, but not the contralateral hindpaw threshold responses were observed at BL (F_7,40_ = 2.27, *P* = 0.048), these differences may simply be due to an exceptionally small variance in the threshold responses of female mice compared to males. However, these differences are not considered physiologically meaningful, as such variance was not observed previously or routinely in either the ipsilateral or contralateral hindpaws. Compared to sham-operated animals, male and female mice with CCI developed clear allodynia through Day 10 [Figure 2B]. A main effect of time (ipsilateral: F_2.22,88.85_ = 265.36, *P* < 0.001; contralateral: F_2.11,84.20_ = 213.38, *P* < 0.001) and surgery (ipsilateral: F_1,40_ = 1612.46, *P* < 0.001; contralateral: F_1,40_ = 978.01, *P* < 0.001), and an interaction between time and surgery (ipsilateral: F_2.22,88.85_ = 240.45, *P* < 0.001; contralateral: F_2.11,84.20_ = 2 0 0.82, *P* < 0.001) was observed. Following BIRT377 treatment, while sham animals remained stably responsive and close to BL thresholds throughout the timecourse, partial bilateral reversal from allodynia was observed by 24 h in males, but not females. Additionally, maximal effects of BIRT377 on allodynia were observed a full day sooner in males than in females [Figure 2B]. Main effects of time (ipsilateral: F_3,120_ = 65.14, *P* < 0.001; contralateral: F_3,120_ = 71.58, *P* < 0.001), injection (ipsilateral: F_1,40_ = 218.80, *P* < 0.001; contralateral: F_1,40_ = 306.81, *P* < 0.001), and surgery (ipsilateral: F_1,40_ = 1818.98, *P* < 0.001; contralateral: F_1,40_ = 1816.36, *P* < 0.001), and interactions between time and sex (ipsilateral: F_3,120_ = 5.31, *P* = 0.002; contralateral: F_3,120_ = 2.86, *P* = 0.040), time and injection (ipsilateral: F_3,120_ = 62.77, *P* < 0.001; contralateral: F_3,120_ = 69.52, *P* < 0.001), and sex and injection (ipsilateral: F_1,40_ = 16.08, *P* < 0.001; contralateral: F_1,40_ = 10.70, *P* = 0.002) were observed. However, by Day 3 post-injection, both males and females achieved similar levels of reversal from allodynia.

### Characterization of sciatic nerve anti- and proinflammatory cytokine/chemokine mRNA levels in males and females following BIRT377 treatment

**Figure 3.**
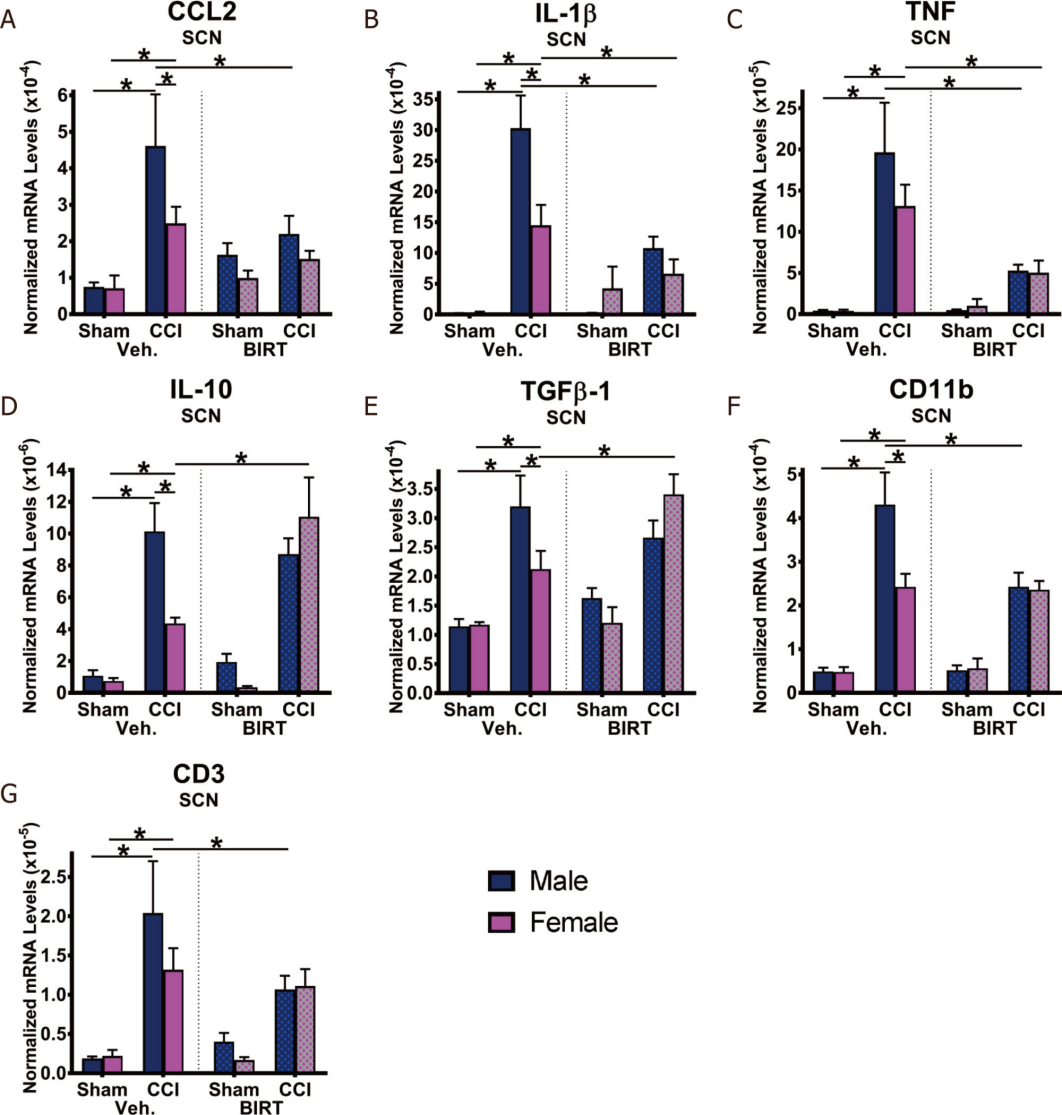
BIRT377 treatment reduced pro-inflammatory cytokine/chemokine in males and females and increased anti-inflammatory cytokines only in females around the injured sciatic nerve. Ipsilateral sciatic nerves were collected from behaviorally verified mice as represented in Figure 2B. (A) Sciatic nerve damage (CCI) induced a significant increase in CCL2 mRNA expression (F_1,1_ = 16.33, *P* = 0.0002), which was greater males than females (F_1,1_ = 4.36, *P* = 0.043). BIRT377 treatment reduced CCL2 mRNA expression in male mice with CCI (F_1,1_ = 7.43, *P* = 0.009). (B) CCI increased IL-1β mRNA expression (F_1,1_ = 54.46, *P* < 0.0001), which was greater in males than females (F_1,1_ = 9.68, *P* = 0.003). BIRT377 treatment reduced IL-1β mRNA levels in mice with CCI (F_1,1_ = 16.3, *P* = 0.0002). (C) Similarly, after CCI, TNF mRNA expression was elevated (F_1,1_ = 35.85, *P* < 0.0001). BIRT377 treatment reduced TNF mRNA expression in mice with CCI (F_1,1_ = 11.53, *P* = 0.001). (D) Following CCI, IL-10 mRNA was dramatically increased (F_1,1_ = 83.79, *P* < 0.0001). BIRT377 treatment further induced IL-10 mRNA expression in females, as a significant interaction between BIRT377 treatment and sex (F_1,1_ = 4.35, *P* = 0.04) was observed. (E) CCI induced an increase in TGF-β1 mRNA expression (F_1,1_ = 52.7, *P* < 0.0001). BIRT377 treatment further increased TGF-β1 in female s with CCI (F_1,1_ = 6.98, *P* = 0.012). (F) After CCI, CD11b mRNA levels were increased (F_1,1_ = 104.2, *P* < 0.0001). Following CCI, males displayed greater levels of CD11b mRNA than females (F_1,1_ = 4.58, *P* = 0.038). BIRT377 treatment reduced CD11b mRNA levels in male mice with CCI (F_1,1_ = 4.884, *P* = 0.032) but not females, as a main effect of sex was observed for CD11b mRNA levels (F_1,1_ = 4.22, *P* = 0.046). (G) CD3 mRNA levels were elevated in mice with CCI (F_1,1_ = 34.3, *P* < 0.0001). Post hoc comparisons revealed that BIRT377 treatment reduced CD3 levels in males with CCI (*P* = 0.016). *p values from post hoc comparisons ranges from *P* = 0.039 to *P* < 0.0001. *n* = 5 in female Sham + Veh and CCI + Veh for TGF-β1 data. *n* = 6 per group unless otherwise indicated

Prior studies suggest contralateral allodynia referred to as “mirror pain” corresponds to pathological events at the spinal cord^[22,72,73,76–81]^. In the current study, inflammatory cytokine changes were examined in the ipsilateral SCN and DRGs, as well as in both the ipsilateral and contralateral LSC dorsal horn to complement prior reports. In the ipsilateral SCN, mRNA levels of the proinflammatory cytokines, CCL2, IL-1β and TNF, were robustly elevated in both males (CCL2, IL-1β and TNF: *P* < 0.0001) and females (CCL2: *P* = 0.039; IL-1β: *P* = 0.0007; TNF: *P* = 0.0006), compared to the corresponding sham-treated controls [Figure 3A-C]. A greater magnitude of CCL2 (*P* = 0.015) and IL-1β (*P* < 0.0002) increase was observed in CCI + Veh males when compared to CCI + Veh females. Treatment with BIRT377 in CCI-operated mice (CCI + BIRT) revealed a reduction in CCL2 in males (*P* = 0.006), and in both males and females, a reduction in IL-1β (male: *P* < 0.0001; female: *P* = 0.049) and TNF (male: *P* = 0.0001; female: *P* = 0.022) mRNA levels, with the largest magnitude of changes observed in males. mRNA levels of the anti-inflammatory cytokines, IL-10 (male: *P* < 0.0001; female: *P* = 0.034) and TGF-β1 (male: *P* = 0.0001; female: *P* = 0.046) were increased in CCI + Veh mice compared to sham-operated conditions (Sham + Veh) [Figure 3D and E]. These data reflect the predicted peri-sciatic anti-inflammatory compensatory response to control ongoing inflammation at the injured SCN ^[47,51]^. Neuropathic females had lower levels of IL-10 mRNA than their male counterparts (CCI + Veh: *P* = 0.001). Interestingly, while BIRT377 treatment in neuropathic males did not induce further increases in these anti-inflammatory cytokines, notable mRNA increases in both IL-10 (*P* = 0.002) and TGF-β1 (*P* = 0.006) were measured in females [Figure 3D and E]. Additionally, because low-level cytokine/chemokine expression remains unaltered in sham-surgery animals given BIRT377 (Sham + BIRT), these data suggest a permissive effect of BIRT377’s action in and around activated peripheral immune cells that have already migrated to the local site of injury in the female SCN microenvironment.

To confirm the presence of the monocytes/macrophages and T cells, which are well-characterized to produce CCL2, IL-1β, TNF, IL-10 and TGF-β1, mRNA levels for CD11b (pan myeloid cell marker) and CD3 (pan T cell marker) were evaluated [Figure 3F and G]. While all neuropathic mice (CCI + Veh) reveal significant increases in peri-sciatic CD11b (male: *P* < 0.0001; female: *P* = 0.0001) and CD3 (male: *P* < 0.0001; female: *P* = 0.005) mRNA levels, reflecting that these peripheral immune cells have migrated to the damaged SCN, the magnitude of increase was greater in males than females for CD11b (*P* = 0.0002). Sham animals treated with BIRT377 did not result in alterations of CD11b and CD3 mRNA levels. However, BIRT377 did significantly reduced mRNA levels of CD11b (*P* = 0.0002) and CD3 (*P* = 0.016) in neuropathic males. These data indicate that by 4 days following i.v. BIRT377 injection, a reduction in both monocyte/macrophage and T cell recruitment around the injured nerve occurred in males. Importantly, these data demonstrate that despite similar levels of CD11b and CD3 mRNA in CCI + Veh and CCI + BIRT females, TNF and IL-1β are reduced, indicating that BIRT377 alters functional responses of immune cells previously recruited around the SCN in females. That is, BIRT377 may be exerting actions on immune cells beyond simply preventing leukocyte trafficking. Given the observed elevation in anti-inflammatory IL-10 and TGF-β1 mRNA levels and reduction in proinflammatory cytokines discussed above, BIRT377 may be dampening the degree of peripheral “damage” signals relayed from the peripheral nervous system to the central nervous system (CNS).

### Sex differences observed in the effects of BIRT377 on reduced mRNA levels of T cell-specific pro- and anti-inflammatory responses

**Figure 4.**
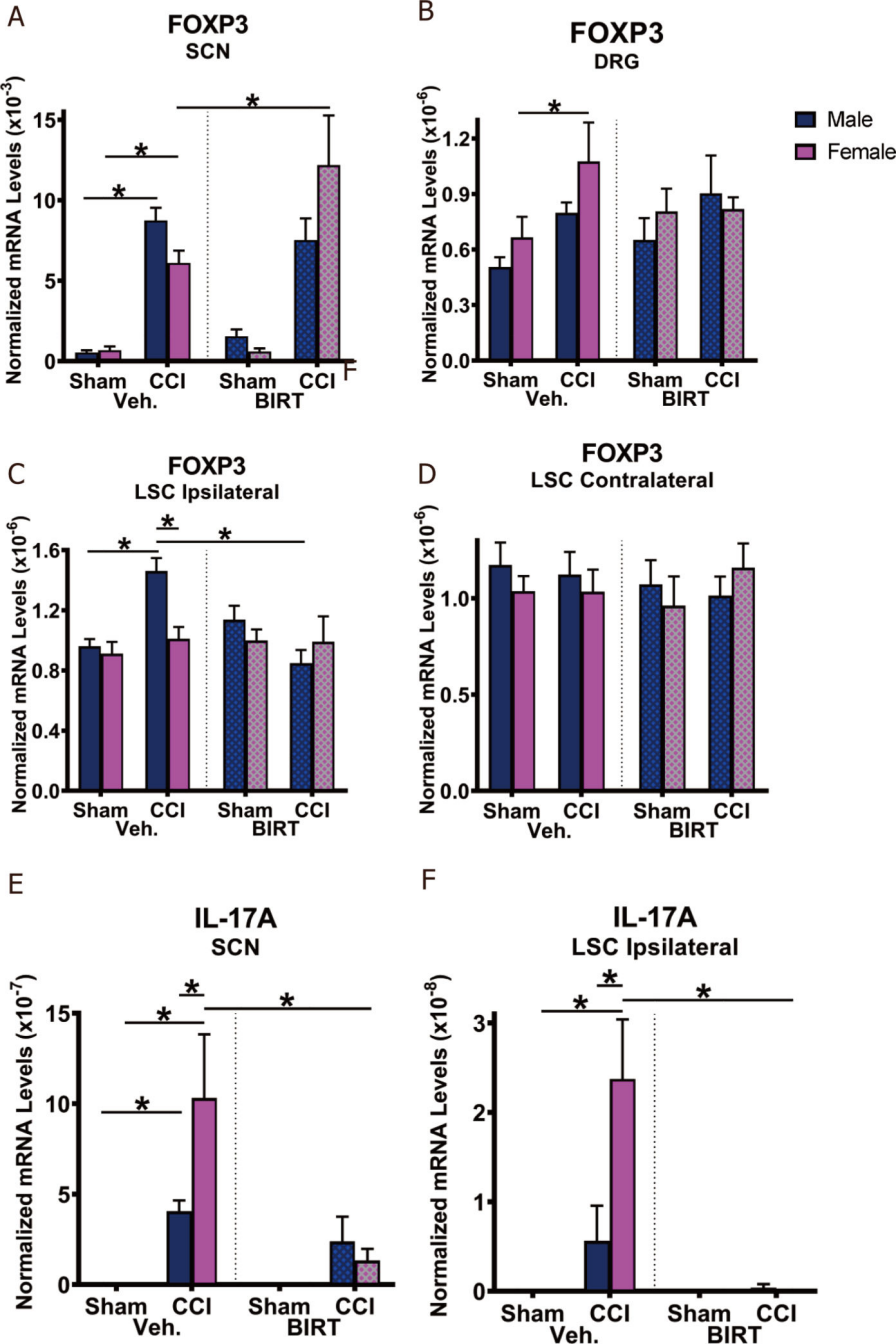
Sex differences in anti-inflammatory FOXP3 and proinflammatory IL-17A from damaged sciatic nerve and in spinal cord. Tissues were collected from behaviorally verified mice as represented in Figure 2B. (A) From ipsilateral sciatic nerve (SCN), CCI induced a significant increase in FOXP3 mRNA expression (F_1,1_ = 76.18, *P* < 0.0001), with CCI-treated females responding to BIRT377 treatment to a greater degree than males (F_1,1_ = 5.38, *P* = 0.025). (B) In the ipsilateral DRGs, FOXP3 mRNA levels were elevated following CCI (F_1,1_ = 6.55, *P* = 0.014), post hoc comparisons revealed significant increases in FOXP3 mRNA levels in females with CCI compared to the sham controls. (C) In the ipsilateral lumbar spinal cord (LSC) dorsal horn, post-CCI induction in FOXP3 mRNA levels was observed in males (*P* = 0.0006). Following CCI, males displayed significantly greater FOXP3 mRNA levels than in females (*P* = 0.001). BIRT377 treatment reduced FOXP3 in males with CCI (*P* < 0.0001), a significant interaction between surgery, injection, and sex (F_1,1_ = 6.5, *P* = 0.014) was observed. (D) In the contralateral dorsal horn, FOXP3 levels were comparable between groups. (E) Post-CCI IL-17A mRNA levels were significantly elevated at the ipsilateral sciatic nerve (F_1,1_ = 21.93, *P* < 0.0001). BIRT377 treatment reduced post-CCI IL-17A mRNA levels (F_1,1_ = 21.93, *P* < 0.0001), with post hoc comparisons revealing a significant reduction of IL-17A mRNA levels following BIRT377 treatment in females with CCI. (F) In the ipsilateral dorsal horn, post-CCI IL-17A mRNA levels were elevated (F_1,1_ = 14.85, *P* = 0.0004). BIRT377 treatment reduced IL-17A mRNA levels in mice with CCI (F_1,1_ = 14.07, *P* = 0.0006), which occurred in females to much a greater degree than males (F_1,1_ = 5.71, *P* = 0.02). Post-CCI induction in IL-17A mRNA levels were much greater in females, than in males (F_1,1_ = 5.23, *P* = 0.02). *p values from post hoc comparisons ranges from *P* = 0.04 to *P* < 0.0001. *n* = 5 in female CCI + BIRT data for DRG FOXP3.*n* = 6 per group unless otherwise indicated

Previous reports demonstrate potential differential contribution of T cell-mediated responses in males and females^[3]^. In the current report, the T cell specific factors, FOXP3 (anti-inflammatory-like T cells) and IL-17A (proinflammatory-like T cells) were analyzed in key anatomical regions of the pain pathway following CCI [Figure 4]. A potential cellular source of anti-inflammatory cytokines is from a subset of T cells referred to as Tregs cells^[57,82]^. The transcription factor responsible for generating Treg cells is FOXP3^[57,59]^. Therefore, to identify a possible source of IL-10 and TGF-β1 (demonstrated in Figure 3D and E), the contribution of Treg cells was indirectly explored by examining FOXP3 mRNA levels. Compared to sham treatment, elevated FOXP3 mRNA was observed in SCN of males (*P* < 0.0001) and females (*P* = 0.003) given vehicle. FOXP3 mRNA levels were further elevated from CCI + Veh group following BIRT377 treatment, only in females (*P* < 0.0001) [Figure 4A]. Similarly, DRGs revealed elevated FOXP3 mRNA levels in CCI + Veh females (*P* = 0.034), while no such increases were observed in males [Figure 4B]. However, BIRT377 did not alter basal FOXP3 mRNA levels in DRG in sham or CCI groups. These data indicate a modest recruitment of Tregs in female DRGs. In contrast to effects observed in females of peripheral tissues (SCN and DRG), only males revealed changes in FOXP3 mRNA levels in the ipsilateral spinal cord, with no FOXP3 mRNA changes observed in the contralateral spinal cord [Figure 4C and D]. Th17-specific proinflammatory cytokine, IL-17A, increases were detected in SCN of both neuropathic males (*P* = 0.04) and females (*P* < 0.0001), when compared to sham-surgery groups [Figure 4E]. Neuropathic (CCI + Veh) females displayed about twice as much upregulation of IL-17A than neuropathic males (*P* = 0.002). These data suggest that Th17 cells may play a more prominent role in females with peripheral neuropathy. This is further supported by BIRT377-mediated reduction of IL-17A mRNA levels in SCNs of pain-reversed females (*P* < 0.0001). Spinal IL-17A mRNA transcripts were absent under non-neuropathic sham-treated conditions in males and females [Figure 4F]. Compared to sham controls, large increases in LSC IL-17A mRNA levels were observed ipsilaterally in neuropathic females (*P* < 0.0001), with modest increases in IL-17A in neuropathic males [Figure 4F]. IL-17A was not reliably detected in the DRGs or contralateral spinal cord samples in any groups. BIRT377 treatment abolished ipsilateral spinal IL-17A mRNA levels in neuropathic females (*P* < 0.0001) and, to a lesser extent, in neuropathic males. These data, along with the data presented in Figure 3G suggest that though there was no difference in overall content of the T cell (CD3 mRNA) population, the quality and differentiation status of these T cells varied in neuropathic animals. These data also show that the actions of BIRT377 on these differentiated T cell subsets is most pronounced in females and may reflect a phenotypic change from proinflammatory to anti-inflammatory, rather than simply reflecting a suppression of T cell recruitment.

### BIRT377 treatment exerts sex-dependent differential effects on T cell differentiation and functional responses

**Figure 5.**
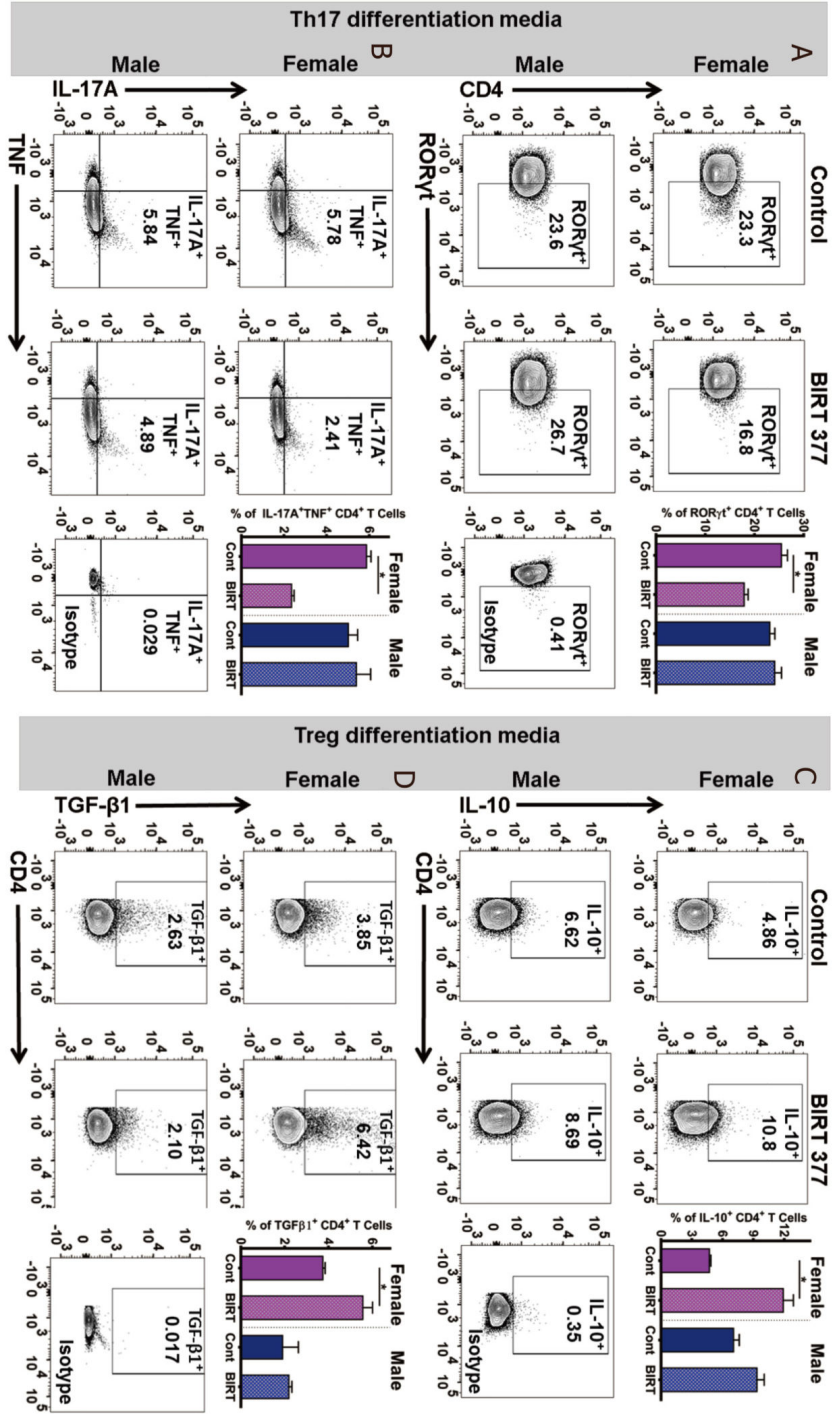
Flow cytometric characterization of *ex vivo* T cells: sex differences in the anti-inflammatory response to BIRT377. Naïve CD4 T cells were conditioned with (A-B) Th17 or (C-D) Treg inducing cytokines, with or without BIRT (500 ng/mL). After 4 days, all viable CD4 T cells were identified and analyzed for the expression of: (A) RORγt (major transcription factor for Th17 cells) or (B) intracellular levels of pro-inflammatory cytokines IL-17A and TNF. BIRT377 treatment reduced RORγt^+^ CD4 T cells (F_1,8_ = 17.99, *P* = 0.002) and IL-17A protein production (F_1,8_ = 24.3, *P* = 0.001) in females. (C-D) From Treg inducing culture, all viable CD4 T cells were analyzed for intracellular levels of (C) TGF-β1 and (D) IL-10. BIRT377 treatment increased intracellular TGF-β1 (F_1,5_ = 12.85, *P* = 0.015) and IL-10 (F_1,6_ = 10.57, *P* = 0.017) protein levels in females. (A-D) Representative flow cytometry plots are shown. Numbers represent the percentages of the (A) RORγt or (B-D) cytokine positive CD4 T cells, where total CD4 T cells are taken as 100%. Corresponding isotype controls (stained with IgG, IgG2a or IgG2b fluorochrome conjugated antibody) for the intracellular staining are shown. Each experimental condition was run in 2–3-well replicates. Error bars represent variations in the well replicates. Data are representative of two independent experiments where T cells were pooled from *n* = 5 males or *n* = 5 females in each experiment. Viable cells were identified based on their light scatter properties (forward and side scatter plot) and viability dye staining. Viable cells were then gated for CD4 cell surface expression; only CD4^+^ T cells were included for further analysis. Positive staining for transcription factor and/or cytokines were determined based on staining with fluorochrome conjugated isotype controls (negative controls). *P values from post hoc comparisons ranges from *P* = 0.014 to *P* = 0.0005

While BIRT377-mediated reduction of IL-17A is indicative of effects of BIRT377 on CD4 T cell differentiation and function, these effects may also be due to the indirect effects of a general reduction in proinflammatory cytokine production (such as TNF) from monocytes that promote Th17 differentiation^[83]^. Therefore, to examine the direct actions of BIRT377 on CD4 T cell differentiation and function, CD4 naïve T cells were given conditioned media to induce the generation of either a Treg or Th17 phenotype, in the presence of control (media only) or BIRT377. Subsequently, the proportion of T cells positive for RORγt^+^ (transcription factor required for the generation of Th17 cells), was analyzed. BIRT377 only reduced the generation of RORγt^+^ T cells in females (*P* = 0.0007), but not in males [Figure 5A]. Additionally, CD4 T cells that are IL-17A^+^, and also produce TNF, were examined as an indication of their functional proinflammatory capacity. Compared to conditioned media alone, the population of IL-17A^+^TNF^+^ CD4^+^ T cells was substantially reduced by BIRT377 exposure only in CD4^+^ T cells derived from females (*P* = 0.001), but not males [Figure 5B]. Furthermore, Treg generation and function in the presence of BIRT377 was examined. Fully differentiated Tregs exert their immune suppressive actions by producing the characteristic anti-inflammatory cytokines, IL-10 and TGF-β1^[57]^. Therefore, the expressions of IL-10 and TGF-β1 proteins were examined as direct evidence of the fully differentiated functional Treg cells. Given that FOXP3 drives Treg generation concurrent with IL-10 and TGF-β1 production, FOXP3 expression was considered redundant. During Treg differentiation in the presence of BIRT377, a large increase in the production of the IL-10 (*P* = 0.0005) and TGF-β1 (*P* = 0.014) was observed [Figure 5C and D] in female-derived pooled T cells, while BIRT377-induced changes in these anti-inflammatory cytokines were absent in male derived T cells. While a trend of increased IL-10^+^ CD4^+^ T cells was also observed in males [Figure 5C], these data demonstrate that female T cells are much more responsive to BIRT377-mediated modulation of pro- and anti-inflammatory T cell-related cytokines. Therefore, BIRT377 regulates one aspect of T cell differentiation and function more readily in females under pathological conditions, which may provide a mechanism for the IL-17A reduction reliably detected only in females at the SCN [Figure 4].

### BIRT377 modulates the proinflammatory cytokine milieu in the DRGs to favor pain reversal

**Figure 6.**
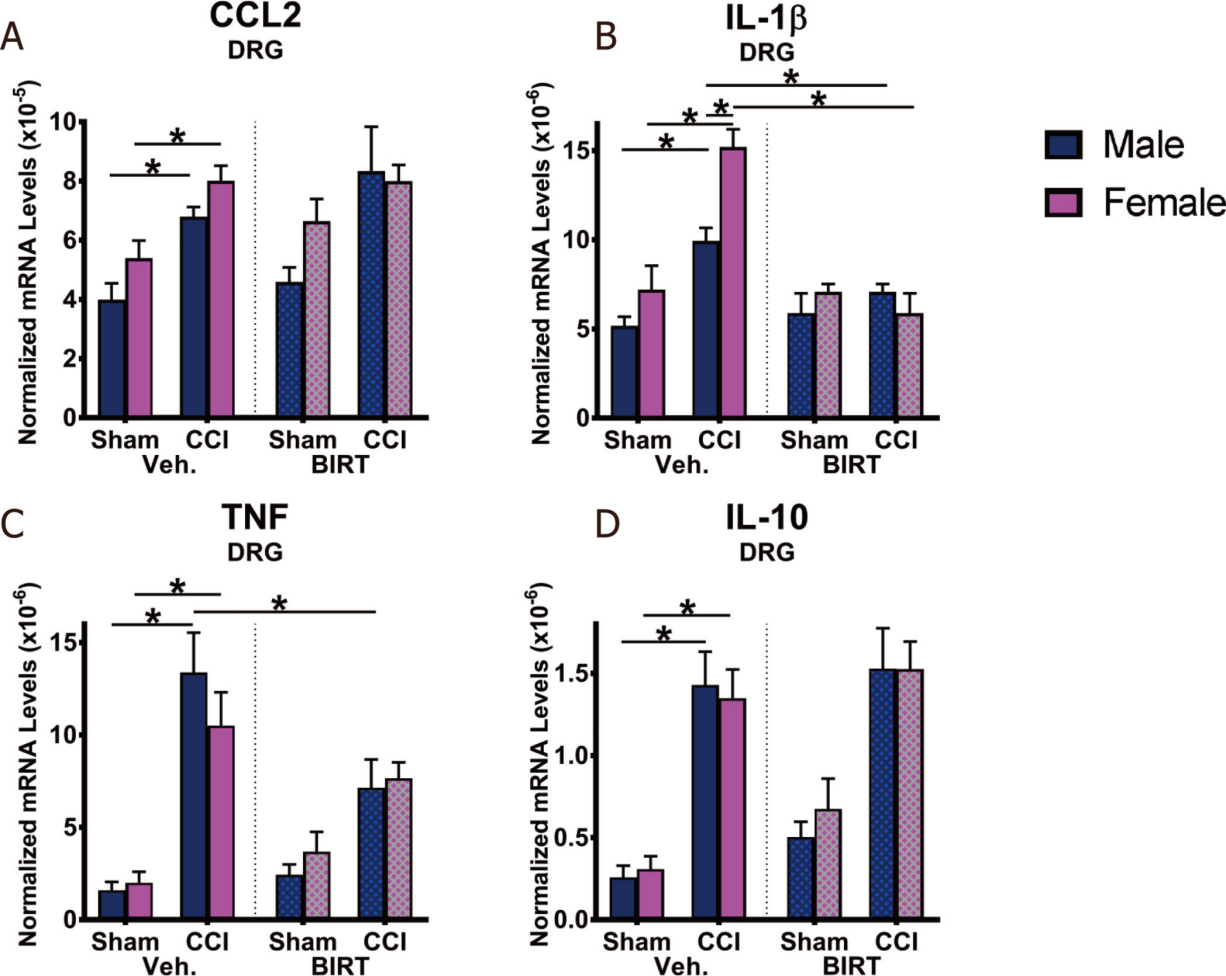
DRGs from males and females reveal similarly reduced IL-1β and TNF mRNA levels following BIRT377 treatment. Total RNA was isolated from ipsilateral lumbar DRGs from the same mice used in Figure 2B, and analyzed for inflammatory cytokines. (A) In the DRGs, CCI induced CCL2 mRNA levels (F_1,1_ = 25.5, *P* < 0.0001) remained unchanged following BIRT377 treatment. (B) CCI increased IL-1β mRNA expression in females (F_1,1_ = 25.4, *P* < 0.0001) to a much greater degree than in males (F_1,1_ = 8.3, *P* = 0.006). BIRT377 treatment reduced IL-1β mRNA levels in mice with CCI (F_1,1_ = 25.4, *P* < 0.0001), with a greater magnitude in females (F_1,1_ = 4.95, *P* = 0.031). (C) Post-CCI TNF mRNA expression levels were increased (F_1,1_ = 61.81, *P* < 0.0001). BIRT377 treatment reduced TNF mRNA expression levels in mice with CCI (F_1,1_ = 9.92, *P* = 0.003). Post hoc comparisons revealed a significant reduction of TNF mRNA levels following BIRT377 treatment in CCI-treated males. (D) Following CCI, IL-10 mRNA expression levels were increased (F_1,1_ = 77.99, *P* < 0.0001). BIRT377 treatment did not further elevate IL-10 mRNA levels during neuropathy. *P values from post hoc comparisons ranges from *P* = 0.029 to *P* < 0.0001, *n* = 6 per group

The most widely examined proinflammatory cytokines known to be critical for pain processing were examined in the ipsilateral DRGs. As predicted, neuropathic male and female (CCI + Veh) mice revealed increases in CCL2 (male: *P* = 0.01; female: *P* = 0.016), IL-1β (males: *P* = 0.0005; female: *P* < 0.0001), TNF (*P* < 0.0001: both sexes). In support of prior reports, a compensatory elevation in anti-inflammatory IL-10 (*P* < 0.0001: both sexes) mRNA levels in the ipsilateral DRGs as compared to Sham + Veh was also measured [Figure 6]. While CCL2 mRNA levels were increased in males and females following CCI, BIRT377 did not alter CCL2 mRNA levels under either condition, and sex differences were not observed. However, a reduction in IL-1β (male: *P* = 0.029; female: *P* < 0.0001) and TNF (males: *P* = 0.001) mRNA levels were measured in allodynic-reversed mice given BIRT377. Unexpectedly, no further increases in IL-10 mRNA levels were observed in BIRT377-treated allodynic-reversed mice. It is notable that the magnitude of IL-1β increase was greater in female CCI + Veh mice (*P* = 0.0002) than males. Correspondingly, the magnitude of BIRT377-induced decreases in IL-1β was greatest in female CCI + BIRT mice [Figure 6B]. In general, the effects of BIRT377 on these pro- and anti-inflammatory cytokines revealed similar trends in both male and female DRGs. Together, these data support that BIRT377 not only affects immune cells at the nerve injury, but also is able to modulate immune cells locally in the DRGs thereby dampening the proinflammatory environment contributing to pain reversal.

### BIRT377 predominantly restores IL-10 levels in the dorsal spinal cord

**Figure 7.**
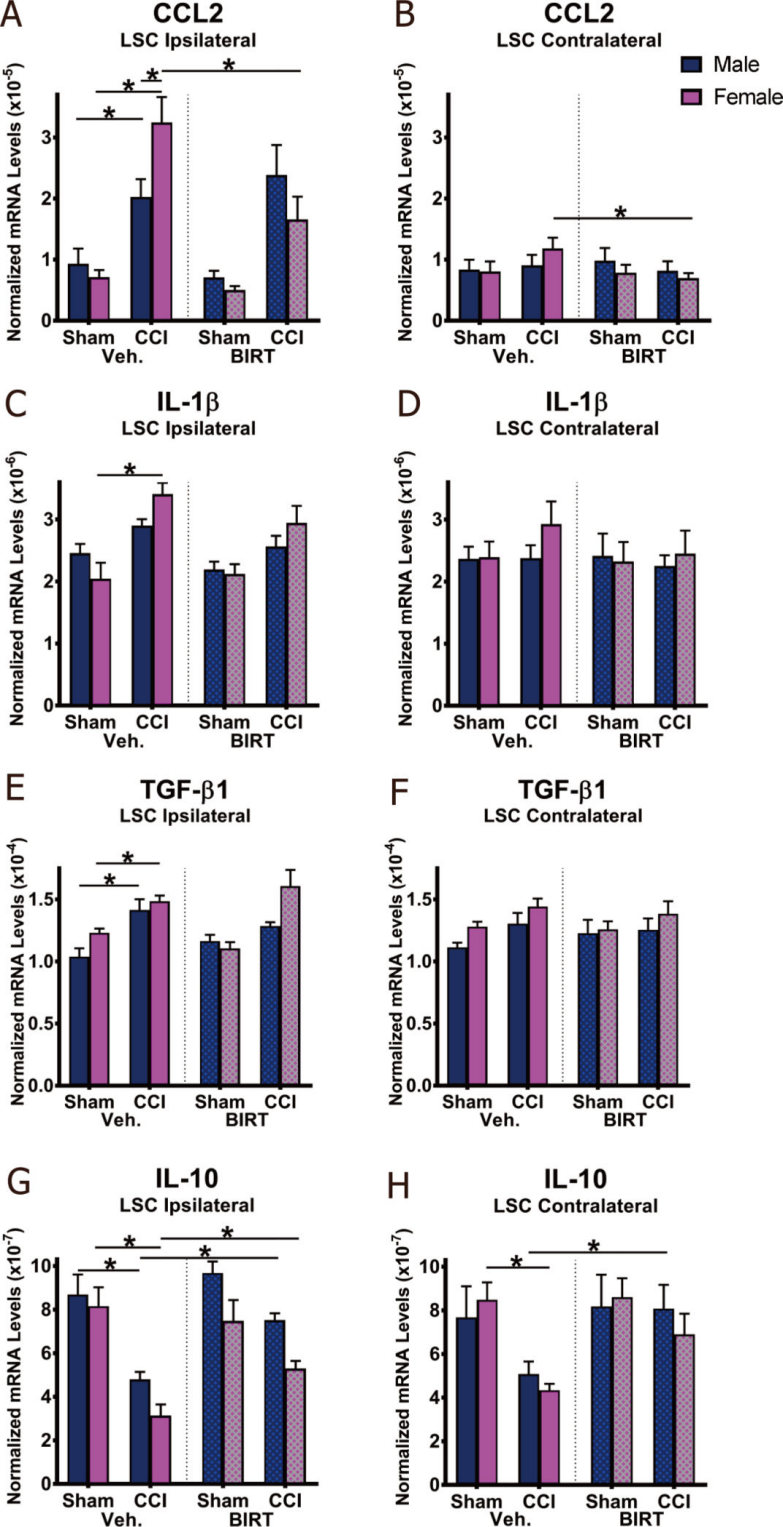
Ipsilateral dorsal spinal cord mRNA levels reveal BIRT377 treatment reduces CCL2 in sciatic damaged females only, while no sex differences occur in elevated IL-10. Lumbar spinal cord (LSC) tissues from behaviorally verified mice (Figure 2B), were collected and analyzed for pain-relevant cytokines. (A-B) Ipsilateral CCL2 mRNA levels were increased following CCI (F_1,1_ = 57.3S, *P* < 0.0001). BIRT377 treatment reduced CCL2 mRNA expression in females with CCI (F_1,1_ = 5.28, *P* = 0.026). Neuropathic females displayed significantly more CCL2 mRNA levels than in neuropathic males (*P* = 0.006). Post hoc comparisons revealed significant reduction in contralateral CCL2 mRNA levels in CCI-treated females following BIRT377 treatment. (C-D) Ipsilateral IL-1β mRNA levels were increased following CCI (F_1,1_ = 32.8, *P* < 0.0001) that occurred in females to much a greater degree than males (F_1,1_ = 6.86, *P* = 0.012). (E-F) While sciatic nerve CCI induced a bilateral elevation in TGF-β1 mRNA levels in both males and females (ipsilateral: F_1,1_ = 40.95, *P* < 0.0001; contralateral: F_1,1_ = 5.23, *P* = 0.027), the magnitude of TGF-β1 mRNA increase following BIRT377 treatment was greater in ipsilateral LSC from females than males (F_1,1_ = 6.56, *P* = 0.014). No differences in TGF-β1 mRNA levels from contralateral LSC were revealed following BIRT377 treatment. (G-H) IL-10 mRNA levels were decreased following CCI (ipsilateral: F_1,1_ = 52.26, *P* < 0.0001; contralateral: F_1,1_ = 9.45, *P* = 0.004). BIRT377 treatment increased IL-10 mRNA expression levels in mice with CCI (ipsilateral: F_1,1_ = 6.19, *P* = 0.017; contralateral: F_1,1_ = 4.94, *P* = 0.032). *P values from post hoc comparisons ranges from *P* = 0.04 to *P* < 0.0001. *n* = 5 in male Sham + Veh and CCI + BIRT for IL-10 contralateral data. *n* = 6 per group unless otherwise indicated

It is possible that BIRT377-mediated changes in cytokine mRNA levels at the damaged SCN and the DRG together influence the inflammatory signals ultimately relayed to the spinal cord dorsal horn where critical pain relays can be facilitated by spinal glial and resident immune cells. Moreover, it is reasonably possible that BIRT377 additionally modulates leukocyte adhesion and spinal trafficking, thereby controlling the peripheral leukocyte milieu recruited to the spinal cord as a consequence of nerve injury. Therefore, spinal mRNA levels of CCL2 was assessed, as CCL2 is a well-established chemokine released from damaged neurons that signals to circulating leukocytes (macrophages as well as subsets of T cells) facilitating immune cell migration to the spinal cord. As predicted, a significant induction of CCL2 mRNA was observed in the dorsal horn of the LSC ipsilateral to the SCN lesion both in males (*P* = 0.014) and females (*P* < 0.0001), with a trend toward increased CCL2 in LSC contralateral to the SCN lesion in CCI females compared to Sham conditions [Figure 7A and B]. Interestingly, CCL2 mRNA levels were similar in neuropathic males given vehicle or BIRT377, whereas a significant bilateral reduction of CCL2 was observed in females given BIRT377 (LSC ipsilateral: *P* = 0.0006; LSC contralateral: *P* = 0.04) [Figure 7A and B]. In support of prior reports documenting the crucial role of IL-1β actions in mediating allodynia, a small but significant increase in the levels of IL-1β mRNA were observed in female CCI + Veh (*P* < 0.0001), with a similar trend observed from the contralateral side. Unexpectedly, BIRT377 treatment did not change IL-1β mRNA levels in the spinal cord [Figure 7C and D].

Anti-inflammatory cytokines TGF-β1 and IL-10 were analyzed in the LSC both ipsilateral and contralateral to the SCN lesion [Figure 7E-H]. Compared to Sham + Veh, a significant induction in ipsilateral TGF-β1 (male: *P* = 0.0004; female: *P* = 0.013) and reduction of IL-10 (male: *P* = 0.0001; female: *P* < 0.0001) mRNA levels were measured in all CCI + Veh mice [Figure 7E and G]. However, following BIRT377 treatment, ipsilateral LSC IL-10 mRNA levels were elevated in both neuropathic males (*P* = 0.005) and females (*P* = 0.02), with similar observations made in female ipsilateral LSC TGF-β1 mRNA levels [Figure 7E and G]. However, the magnitude of IL-10 increases in ipsilateral LSC was greater in CCI + BIRT males than females, and CCI + BIRT females displayed lower IL-10 levels than males (*P* = 0.019). Contralateral IL-10 mRNA levels displayed the same pattern as ipsilateral dorsal horn following CCI: IL-10 mRNA levels were significantly decreased in neuropathic females (*P* = 0.004), along with a similar trend in males (*P* = 0.07), compared to their corresponding Sham + Veh groups. BIRT377 treatment increased contralateral IL-10 significantly from CCI + Veh in males (*P* = 0.04), with a similar trend observed in females (*P* = 0.06) [Figure 7H]. These data indicate that BIRT377-mediated pain reversal corresponds to increased bilateral spinal IL-10 mRNA levels in neuropathic animals of both sexes.

### BIRT377 reduced astrocyte activation in the spinal cord

**Figure 8.**
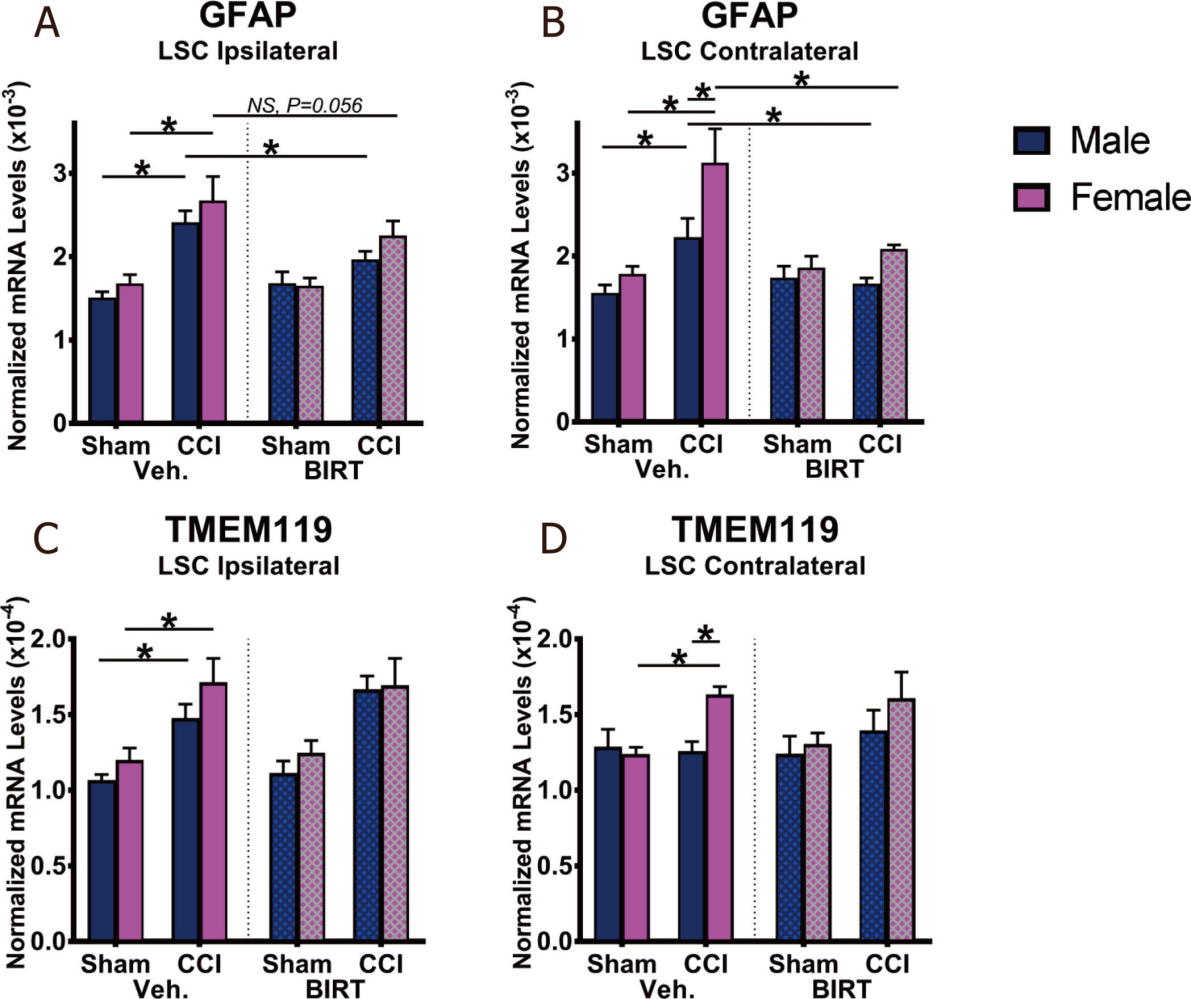
Spinal GFAP mRNA levels are reduced following BIRT377 in both males and females. mRNA was extracted from tissues behaviorally verified in Figure 2B. (A-B) Astrocyte activation marker, GFAP, mRNA levels were increased on both sides of the spinal cord, in all mice with CCI (ipsilateral: F_1,1_ = 41.91, *P* < 0.0001; contralateral: F_1,1_ = 16.68, *P* = 0.0002). BIRT377 treatment reduced spinal GFAP mRNA levels in mice with CCI (ipsilateral: F_1,1_ = 5.533, *P* = 0.023; contralateral: F_1,1_ = 12.28, *P* = 0.001). In the contralateral side, CCI-induced GFAP mRNA levels were greater in females than in males (F_1,1_ = 9.9, *P* = 0.003). (C-D) Microglial proliferation marker, TMEM119, mRNA levels were increased following CCI (ipsilateral: F_1,1_ = 40.49, *P* < 0.0001; contralateral: F_1,1_ = 7.66, *P* = 0.008). Neuropathic females displayed significantly greater contralateral TMEM119 expression than neuropathic males (*P* = 0.015), as a main effect of sex (F_1,1_ = 4.16, *P* = 0.048) was observed. TMEM119 mRNA levels were comparable between BIRT377 or vehicle treated neuropathic males or females bilaterally. No significant difference was detected between male or female Sham + Veh. and Sham + BIRT group. *P values from post hoc comparisons ranges from *P* = 0.04 to *P* < 0.0001. *n* = 6 per group

The data above show that i.v. BIRT377 corresponds to reduced proinflammatory cytokines in both the SCN and DRGs, and reduced CCL2 in the ipsilateral spinal cord, while elevating anti-inflammatory cytokines in the SCN, DRGs, and LSC. Persistent microglial and astrocyte activation in the spinal cord is critical for the chronicity of sciatic neuropathy. Reducing the pro-inflammatory cytokine milieu at peripheral anatomical regions (SCN and DRG) of the pain pathway may likely reduce chronic pain relays to the spinal cord, and in doing so, may reduce spinal glial activation and ultimately, pathological pain processing. In support of this possibility, GFAP (marker of astrocyte activation) and TMEM119 (related to microglial activation) mRNA levels were examined in the ipsilateral and contralateral LSC [Figure 8]. Data revealed that GFAP mRNA levels were significantly increased in the ipsilateral dorsal horn of CCI + Veh animals compared to Sham + Veh animals (male: *P* = 0.0001; female: *P* < 0.0001), in support of prior reports^[26,84]^. Compared to CCI + Veh animals, GFAP mRNA levels of CCI + BIRT males were significantly decreased (*P* = 0.044), with a similar trend (*P* = 0.056) observed in females [Figure 8A]. Similar increases in GFAP mRNA levels were observed in contralateral LSC in CCI + Veh animals (male: *P* = 0.015; female: *P* < 0.0001). Similarly, contralateral GFAP levels returned to BL in all neuropathic mice following BIRT377 treatment (male: *P* = 0.04; female: *P* = 0.0003) [Figure 8B]. Of note, GFAP transcripts from the contralateral side of the spinal cord were significantly greater in neuropathic females than in males (*P* = 0.001). Ipsilateral TMEM119 mRNA levels were also increased, indicative of increased microglial activation following CCI [Figure 8C and D] in both males (*P* = 0.009) and females (*P* = 0.001). An elevation in TMEM119 mRNA levels were also observed from the contralateral spinal cord, but only in females (*P* = 0.011). Surprisingly, BIRT377 treatment did not change TMEM119 mRNA levels from the ipsilateral or contralateral spinal cord in males or females.

### BIRT377 treatment did not result in systemic immune changes

**Figure 9.**
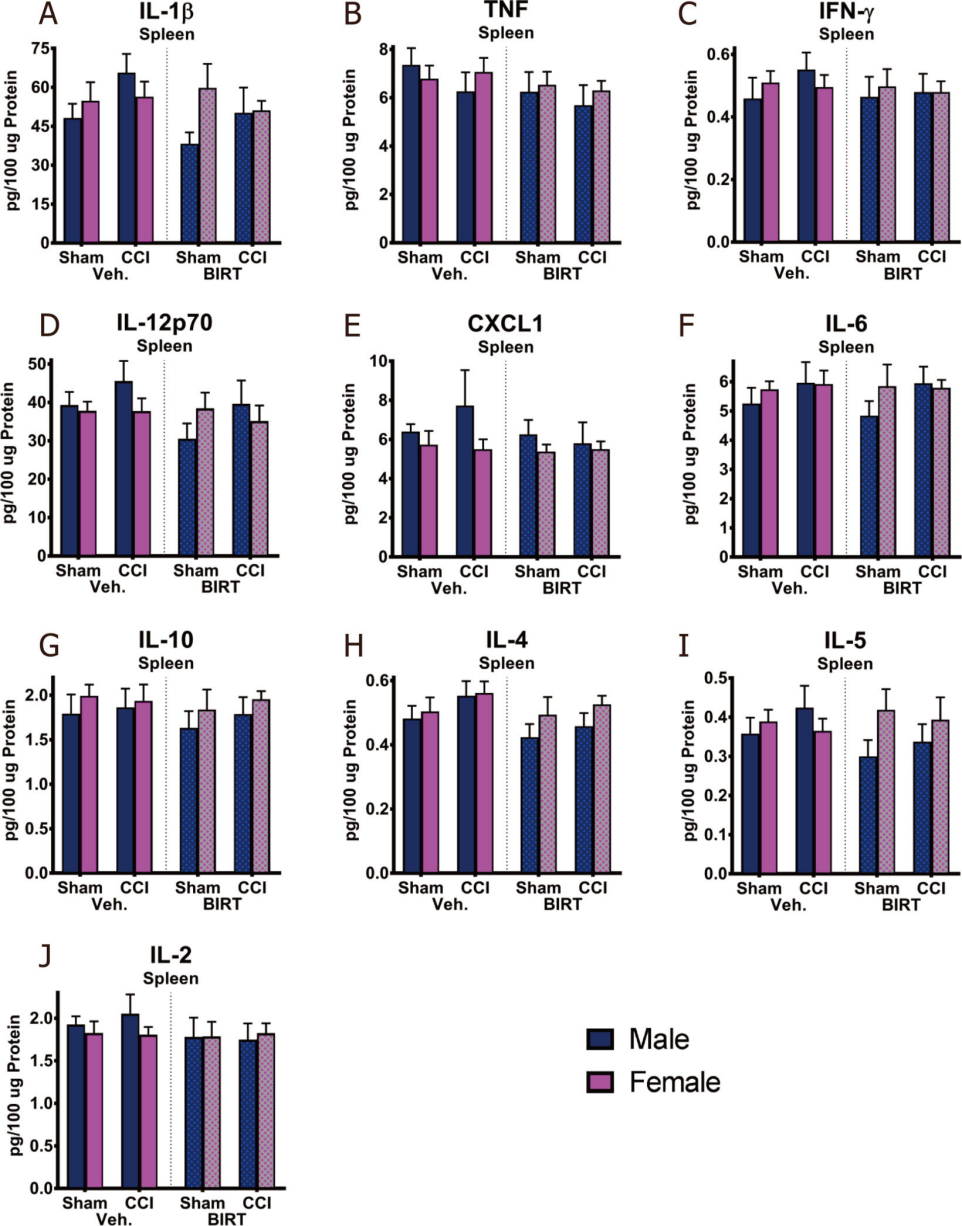
BIRT377 treatment did not result in systemic immune changes. (A-J) Spleens were collected from the same mice used in Figure 2B and analyzed for inflammatory cytokines. Splenic cytokine and chemokine protein levels were similar regardless of surgical manipulation, treatment, or sex. *n* = 6 in each group

Spleens were collected to capture a broad population of peripheral circulating immune cells inclusive of monocytic macrophages, neutrophils, dendritic, and T and B cells. Importantly, all splenic protein data presented [Figure 9A-J] are from behaviorally characterized mice, as demonstrated in Figure 2B. Protein analysis of spleen revealed that, compared to sham treatment, a trend toward increased proinflammatory cytokine IL-1β and chemokine C-X-C motif ligand 1 (CXCL1) occurred in CCI male mice, which returned to basal levels following BIRT377 treatment [Figure 9A and E]. However, comparisons did not reveal statistically significant differences, suggesting that the reservoir of circulating leukocytes, such as monocytes and lymphocytes represented in the spleen cannot act as surrogate indicators of atypical neuroimmune events in key anatomical regions of the pain pathway. It is possible that the immune changes observed in discrete regions involved in the pain pathway (SCN, DRGs and LSC) are exceedingly localized such that detection from the systemic pool of immune cells is not measurable. More likely, these observations may indicate that further immune cell differentiation occurs after their migration to key pain-relevant nervous tissue regions in response to signals from local tissue-damage.

## DISCUSSION

Reports focused on understanding neuroimmune changes when performing a comparative approach between sexes are rare with most studies applying male rodent models^[4,25–27]^. In recent years, published reports provide compelling evidence that activation of spinal microglia play a direct role in generating pathological pain in males, while in females, the actions of T cells are critically important^[3,4]^. Consistent with prior reports^[4,26]^, we find that, while the onset, magnitude and spontaneous reversal of allodynia are similar in males and females [Figure 1], divergent peripheral immune and neuroimmune responses are present during neuropathy. We demonstrate for the first time that during neuropathy, T cell-associated pro- and anti-inflammatory responses in males and females are different at discrete anatomical regions critical in the pain pathway of sciatic neuropathy. During neuropathy, females displayed more profound Th17 specific responses (IL-17A) than males, both at the injured nerve and in the corresponding LSC [Figure 4]. While regulatory T cell (Tregs) recruitment (FOXP3 expression) was evident at the injured SCN in both males and females, only females displayed reliable increases of FOXP3 at the DRGs [Figure 4]. The beneficial role of blocking the active conformational state of LFA-1 was demonstrated in both sexes [Figure 2] by reducing immune cell accumulation in damaged SCN [Figure 3]. However, BIRT377 modulated T cell function in a sex-specific manner [Figure 5]. For example, T cells from females were significantly more responsive to the anti-inflammatory effects of BIRT377 [Figure 5]. Similarly, BIRT377 treatment elevated T cell-associated anti-inflammatory factors (FOXP3, IL-10 and TGF-β1) and reduced the proinflammatory T cell cytokine, IL-17A in the peri-sciatic milieu, predominantly in neuropathic females [Figures 3 and 4]. Interestingly, despite the fact that there was no additional change in FOXP3 expression in the LSC, the profound reduction of IL-17A in the LSC in i.v. BIRT377 treated females indicates a limited role exists for spinal Treg actions on pain reversal. Importantly, a reduction of peri-sciatic IL-17A co-occurs with profound spinal cord suppression of IL-17A, suggesting the excitatory input from centrally projecting nerve terminals into the lumbar spinal region ultimately leads to a reduction in proinflammatory factors that includes IL-17A. While these data demonstrate that a potential role for spinal IL-17A in pro-nociceptive signaling occurs, it remains unclear whether IL-17A acts in concert with other well-characterized spinal proinflammatory factors, or whether IL-17A is a necessary factor in pain signaling. Therefore, the results from the current data provide the rationale for performing future studies to examine whether specifically blocking the spinal actions of IL-17A also suppresses allodynia from peripheral neuropathy. While these additional studies would aid in understanding the role of IL-17A in chronic neuropathic pain, the current data are the first documented evidence that a reduction in lumbar spinal IL-17A expression co-occurs with a reduction in allodynia from CCI in both males and females [Figure 4]. Strikingly, peripheral BIRT377 reduced spinal astrocyte activation, but had little impact on microglial activation [Figure 8]. Overall, BIRT377 created an anti-inflammatory bias in discrete regions along the pain pathway of the CCI model in both sexes [Figures 3–7], thereby contributing to pain reversal. A brief summary of immune changes during CCI-induced neuropathy and BIRT377-mediated effects are listed in Table 1. This initial comparative analyses of glial/myeloid and T cell-related cytokines and their corresponding transcription factors that are altered by preventing β2-integrin (LFA-1) signaling, provides insight into possible mechanisms leading to peripheral sciatic neuropathy between males and females.

### Sex differences in peripheral inflammatory reactions to nerve injury

Remarkable sex differences of immune system activity are observed in different disease models^[85–89]^. Sex differences in TLR4 responses to pathogen stimulation have been observed, whereby females produce similar or less IL-1β and TNF compared to males^[85]^. Female-derived immune cells are more efficient in antigen presentation and initiating adaptive immune responses^[90]^. In the CCI model, peripheral inflammatory reactions to nerve injury are mediated by endothelial cells of the blood-nerve barrier and Schwann cells (e.g., undergoing myelin degeneration), followed by circulating leukocytes recruited in response to injury^[7]^. CCL2 signaling recruits monocytes, neutrophils and a subset of T cells^[18,91,92]^. We observed greater induction of SCN CCL2, along with greater SCN CD11b levels in males than females during neuropathy. These data, in combination with greater SCN IL-1β production in males suggest greater monocyte/macrophage-driven immune responses in males than females.

Though we detected T cell recruitment in both sexes, the critical finding was in detecting a T cell differentiation bias toward a proinflammatory status that was significantly greater in females than males. Moreover, responses to BIRT377 in females were robustly anti-inflammatory. For example, while an induction of SCN FOXP3 (transcription factor in Tregs for IL-10 and TGF-β1) was detected from both neuropathic males and females, BIRT377 induced additional increases only in SCNs of females with no change in FOXP3 levels in males. Even more striking were the robust levels of SCN IL-17A of neuropathic females compared to males, with profound blunting of IL-17A in pain reversed females relative to pain-reversed males [Figure 4A to F]. These data indicate that females mount stronger proinflammatory T cell responses following nerve injury compared to males despite an abundance of peri-sciatic T cells (as indicated by CD3, global T cell marker) present in both males and females. Moreover, the striking FOXP3 increase and simultaneous IL-17A decrease predominantly in female SCN suggests that BIRT377 favors targeting T cells derived from females than from males.

Interestingly in DRGs, IL-1β levels were greater in neuropathic females. It is possible that the combination of T cell-mediated responses, along with myeloid-driven proinflammatory actions culminate in greater nociceptive factors that induce further hyperexcitability relayed to the spinal cord in females. The fact that reliable induction of FOXP3 was observed only in female DRGs may reflect the anti-inflammatory rebound in response to inflammatory signals. Recruitment of Treg cells could function to control bystander injury-related proinflammatory cytokines.

### Sex convergent and sex divergent aberrant spinal immune responses underlying chronic pain

Despite evidence of microglial activation in neuropathic females^[26]^, microglial TLR4 signaling is only necessary for the development of neuropathy in males, whereas astrocytic signaling under neuropathic conditions is observed in both males and females^[3,25,26]^. Supporting prior observations, we detected astrocytic and microglial activity in both sexes during neuropathy [Figure 8]^[4]^. However, we noticed that induction of ipsilateral CCL2 in conjunction with IL-1β was greater in females [Figure 7]. The reduction of basal IL-10 levels, a finding that our group has previously observed in chronic neuropathic male rats^[93,94]^, appeared more pronounced in neuropathic female than male mice. Note that along with greater astrocyte (as assessed by increased GFAP) and microglial activation (TMEM119) and increased CCL2 in the contralateral side, a simultaneous decrease in IL-10 was measured, indicating that contralateral spinal cord IL-10 expression in females may reflect a greater impact of this cytokine in controlling proinflammatory contralateral glial activation. It is noteworthy that, other than CCL2 in females, changes in injury-related contralateral spinal IL-1β or TGF-β1 were not detectable in males or females, despite ongoing contralateral allodynia. Therefore, the reduction of the basal levels of spinal IL-10, rather than the presence of these specific proinflammatory cytokines, is likely a better indicator of ongoing allodynia.

We speculate that T cell-mediated proinflammatory cytokines (e.g., IL-17A) at the injury site may consequently drive sciatic “damage” signals, leading to the release of factors from nerve terminals in the spinal cord that communicate to pain projection neurons. Astrocytes and microglia local to the dorsal horn of the spinal cord respond to these damage signals from SCN terminals. Though activated astrocytes are capable of producing IL-17A^[95,96]^, contralateral IL-17A was not detected despite astrocyte activation, suggesting that contralateral IL-17A is not a key factor in contralateral glial activation. In fact, the absence of contralateral IL-17 may reinforce the possibility that ipsilateral immune-related signaling may drive contralateral spinal cord pain neuron excitability via astrocyte-specific gap junctional communication^[73,80]^. Interestingly, supraspinal mechanisms such as activation of cortical areas important in pain processing, and descending facilitation from key brainstem areas may contribute in contralateral allodynia as well^[97,98]^. Proinflammatory cytokines in pain related brain regions are capable of impairing descending inhibitory pain pathways^[99]^. Whether, differential immune mechanisms following nerve injury influence the descending pathways involved in manifesting mirror image pain in different sexes would be an interesting avenue for future exploration.

Though astrocytic activation during neuropathy is common in both sexes, it is possible that microglia in males and infiltrating Th17 cells in females are the predominant cell types responsible for driving chronic excitation of astrocytic-neuronal interaction. In support of this possibility, the current report demonstrated a robust upregulation of IL-17A in the spinal cord of neuropathic females. Our prior data indicates the presence of activated T cells (T-bet and RORγt mRNA transcripts, which are critical transcription factors for Th1 and Th17 respectively) in the ipsilateral LSC in neuropathic female rats^[100]^. Therefore, Th17 cells likely infiltrate the ipsilateral spinal cord and interact with astrocytes where ongoing pathology is present, inducing a feed-forward astrocyte-proinflammatory chemokine (e.g., CCL2) and cytokine production ^[101,102]^, as observed in this study. However, re-programming of differentiated T cells and their functional responses can occur in response to the local cytokine milieu in the CNS^[102–104]^. Therefore, the absence of contralateral IL-17A does not prove a lack of T cell recruitment or their actions in contralateral neuropathy.

### Sex-specific mechanistic differences of BIRT377 pain reversal

#### BIRT377-mediated effects on myeloid/glial activation

Numerous reports suggest that blocking LFA-1 actions restricts migration of monocytes and T cells to injured tissues^[37,105]^. Following BIRT377 treatment, both males and females display decreases in peri-sciatic IL-1β and TNF, which are generally myeloid-derived. Reduced CD11b levels around the injured nerve are found only in males. Together, these data suggest BIRT377 may reverse pain in males mainly by blocking myeloid cell migration and consequent exposure to proinflammatory cytokines. However, in females, the effect of blocking the active conformation of LFA-1 by BIRT377 appears to directly alter transcriptional regulation of pro- and anti-inflammatory cytokines of myeloid-derived cells. Previous studies suggest that a lack of LFA-1 interaction with leukocytes increases IL-10, switching macrophage activation from a proinflammatory bias to an anti-inflammatory state^[38,42]^. Therefore, BIRT377-mediated re-programing of myeloid cell function may also occur, and possible sex differences regarding these observations need to be further explored.

#### BIRT377-mediated effects on T cells

Though the exact mechanism(s) are unclear, LFA-1 signaling interacts with T cell activation, and therefore, modulates adaptive immune responses^[44,54]^. While sex was not specified, previous studies suggest that blocking LFA-1 actions decreases Th17 differentiation and increases FOXP3^+^ Tregs^[39,106]^. Consistent with the *in vitro* T cell findings demonstrated in the current report, *in vivo* increases in IL-10 and TGF-β1 were observed along with increases in FOXP3 and reduced IL-17A levels at the SCN only in females following BIRT377 treatment. Therefore, BIRT377 treatment is beneficial for pain reversal by affecting both immune cell migration and modulation of their actions at local sites of inflammation, thereby, indirectly influencing the spinal-immune milieu during neuropathy. Interestingly, for both sexes, BIRT377 did not change microglial activation, suggesting that reducing astrocytic activation and increasing IL-10 levels at the spinal cord is sufficient to reverse allodynia.

We have recently reported that intrathecal (spinal) application of BIRT377 in a rat CCI model leading to chronic neuropathy, dorsal horn spinal astrocyte activation and IL-1β are reduced, as evidenced by immunohistochemical staining and image analysis measures^[107]^. Though BIRT377-mediated effects on mRNA levels of IL-17A, IL-10, and TGF-β1 are supportive of the protein levels of these cytokines [Figure 5], direct quantification of protein levels of all the diverse immune markers would further strengthen the findings in the current report ^[30,32]^, along with semi-quantitative analysis of expression markers using immunohistochemical and image analysis methods that can capture within-region specific changes in comparatively sparse T cell subtypes^[33,108]^.

In conclusion, this study supports the presence of divergent proinflammatory cytokines in males and females following peripheral nerve injury, which has important implications when developing pain therapeutics. Despite the observed cytokine/chemokine and related transcription factor expression differences in SCN, DRG and LSC, systemic blockade of LFA-1 activation is beneficial for pain reversal in both sexes. Therefore, BIRT377 may serve as a novel therapeutic for chronic pain and other CNS diseases.

## Figures and Tables

**Table 1.** A brief summary of changes in immune factors following CCI and BIRT377 treatment

Tissue Regions	Immune Parameters	Male		Female	

		CCI^#^	CCI+ BIRT377^##^	CCI^#^	CCI+ BIRT377^##^
Sciatic Nerve (Ipsilateral)	CCL2	Up	Down	Up^*^	
	IL-1β	Up	Down	Up^*^	Down
	TNF	Up	Down	Up	Down
	IL-10	Up		Up^*^	Up
	TGFβ−1	Up		Up^*^	Up
	CD11b	Up	Down	Up^*^	
	CD3	Up	Down	Up	
	FOXP3	Up		Up	Up
	IL-17A	Up		Up^*^	Down
DRGs (Ipsilateral)	CCL2	Up		Up	
	IL-1β	Up	Down	Up^*^	Down
	TNF	Up	Down	Up	
	IL-10	Up		Up	
	FOXP3			Up	
Lumbar Spinal Cord (Ipsilateral)	CCL2	Up		Up^*^	Down
	IL-1β			Up	
	TGFβ−1	Up		Up	
	IL-10	Down	Up	Down	Up
	FOXP3	Up^*^	Down		
	IL-17A			Up^*^	Down
	GFAP	Up	Down	Up	
	TMEM119	Up		Up	
Lumbar spinal Cord (Contralateral)	CCL2				Down
	IL-1β				
	TGFβ−1				
	IL-10		Up	Down	
	FOXP3				
	GFAP	Up	Down	Up^*^	Down
	TMEM119	Up		Up	

* Fold changes were significantly different in males versus females.

# Comparison between Sham+Veh and CCI+Veh,

## comparison between CCI+Veh and CCI+BIRT377
